# Genome Edited Crops Touch the Market: A View on the Global Development and Regulatory Environment

**DOI:** 10.3389/fpls.2020.586027

**Published:** 2020-10-09

**Authors:** Jochen Menz, Dominik Modrzejewski, Frank Hartung, Ralf Wilhelm, Thorben Sprink

**Affiliations:** Julius Kühn Institute (JKI) – Federal Research Centre for Cultivated Plants, Institute for Biosafety in Plant Biotechnology, Quedlinburg, Germany

**Keywords:** genome editing, regulation, legislation, site directed nucleases, policies, agriculture, modified crops, plant biotechnology

## Abstract

Products of genome editing as the most promising “New Plant Breeding Technology” (NPBT) have made the transition from the lab to the market in a short time. Globally, research activities employing genome editing are constantly expanding and more and more plants with market-oriented traits are being developed, and companies have already released the first genome edited crops to the market. Few countries, most of which are located in the Americas, have adapted legislations to these technologies or released guidelines supporting the use of genome editing. Other countries are debating the path to come either because there is no clarity on the legal classification or due consensus is hampered by a renewed GMO debate. In recent years (2017−2020), eight countries have introduced guidelines clarifying the legal status of genome edited products and many of those are actively committed to international harmonization of their policies. In this publication we give an overview on the current and potentially future international regulatory environment and an update on plants derived by genome editing with market-oriented traits.

## Introduction: Genome Editing

The most progressive step in breeding is techniques that enable a targeted intervention in the genome with or without the integration of a transgene. The “genome editing” techniques comprise a set of methods developed in the recent years to precisely modify genomes of organisms. Genome editing employs variants of site directed nuclease (SDN) technologies and oligonucleotide-directed mutagenesis (ODM). We focus here on the use of genome editing in plant breeding, although the modes-of-action of these techniques are not limited to plants and are applied also in other organisms [for a review see Wang et al. (2016); Gaj et al. (2016)]. Application of genome editing can result in modifications which are identical to those derived from conventional breeding, natural−or induced mutations (Grohmann et al., 2019). In contrast to the older techniques which randomly introduce undirected changes (Sikora et al., 2011), genome editing allows the precise modification of a plants genome similar to a delete-copy-paste-mechanism in a text editor. Four SDN systems have been used so far, besides Zinc-Finger-Nucleases (ZFNs) and Meganucleases, which emerged late in the last century, Transcription Activator like Effector Nucleases (TALENs) and Clustered Regularly Interspaced Short Palindromic Repeat/CRISPR associated proteins (CRISPR/Cas) systems are used. CRISPR/Cas-based systems are currently the most prominent SDN approaches. For all SDNs their mode-of-action is in principle the same: once present in a cell by insertion/expression and or transfection, the SDN is capable of cutting the genome at a targeted site. The cellular DNA-repair mechanisms fix the cut sites either by non-homologous end joining (NHEJ) or by homology directed repair (HDR) (Li and Xia, 2020). As NHEJ can be an error-prone process, insertions or deletions (InDels) can appear at the respective genomic site, which can lead to a loss-of-function edited gene sequence due to frameshift mutations. During repair, the presence of a DNA-template that is largely homologous to the sequence at the target site except for a few base pairs can initiate the plant cells HDR processes to modify the genome sequence according to these differences (van Vu et al., 2020). When cutting the genome at more than one neighboring sites or on different chromosomes, even longer DNA-sequences can be replaced, removed or chromosome fragments can be interchanged. Especially for CRISPR/Cas-systems many varieties and modifications are already known and new variants are being steadily developed. Thus, genome-editing by using SDNs can be categorized in three types:

(1) the induction of single point mutations or InDels (SDN-1),
(2) short insertions or editing of a few base-pairs by an external DNA-template sequence (SDN-2) and,
(3) the insertion of longer strands (SDN-3) of allochtonous (transgenes) or autochtonous sequences (cisgenes).

Among the latest achievements is a base editor, which consists on a fusion of a dead (non-cutting) Cas9 with a nucleotide deaminase, which enables the mutation of a C/G base pair into an A/T pair and vice versa without cutting the genome (Shimatani et al., 2017, 2018; Li et al., 2018; Molla and Yang, 2019; Zhang et al., 2019). Such point mutations we also categorize in SDN-1. The increasing diversity of genome editing methods and approaches thus leads to a broad spectrum of applications in plants that are progressively applied commercially.

The SDN (SDN-1/2/3) terminology has been adopted by many countries to legally categorize SDN applications. Besides SDNs, ODM is another genome editing technique which can be used for the induction of targeted mutations of a single or a few adjacent nucleotides in the genome (Sauer et al., 2016). For ODM single-stranded DNA or chimeric RNA/DNA/LNA hybrids are used, that are homolog to the targeted genome sequence except for the nucleotides to be changed. It is explained that the DNA repair mechanisms recognize these mismatches and induce a miscorrection in the targeted genome sequence. Since for ODM a template is introduced into the cell, the authors consider the outcome of ODM is most comparable to an SDN-2 event.

### Old Laws for New Techniques Provide Room for Uncertainty

Most national and international legislations do not explicitly refer to products of genome editing due to its novelty and diversity of products. Most regulations of biotechnology applications in breeding refer to the use and commercialization of conventional genetically modified organisms (GMOs) and products thereof. Thus for conventional GMOs, the legal status is clear and often in line or similar with the definition given in the Cartagena Protocol an international agreement which aims to ensure the safe handling, transport and use of so called “living modified organisms (LMOs)” resulting from modern biotechnology. The protocol defines a LMO “[…] *any living organism that possesses a novel combination of genetic material obtained through the use of modern biotechnology*” (Secretariat of the Convention on Biological Diversity, 2000) and many national legislations use *somewhat similar* definitions for a GMO. These definitions originate in the years before 2000 when modern biotechnology was mostly considered as insertion or deletion of recombinant DNA in organisms beyond the species border and when most genome editing methods were neither known nor applied. In that period many countries established national legislations on genetic engineering, which are based on the Cartagena definition to assure biosafety of GMO products (see Table 1). Current safety regulations governing import, cultivation and the use of GMOs for food and feed were initiated meeting concerns that genetic engineering potentially generates unforeseen risks for human and animal health and the environment. A generic risk caused by the technology itself has not been proven since more than 30 years of biosafety research (European Commission, 2010; Nicolia et al., 2014; Leopoldina DFG and Akademieunion, 2019). A GMO for cultivation or import for food and feed production needs to pass a rigorous safety assessment in most countries for approval. Breeders or importers must provide extensive data for the safety assessment. Hence, GMO approvals are time and cost intensive in many countries. In the European Union, approval of a GM crop costs approximately between 11 and 17 million Euro and takes on average 6 years (EuropaBio, 2019), but most member states restricted or banned cultivation on their territories due to, *inter alia*, biosafety concerns of stakeholder groups. On European territory, only Spain and Portugal still grow one GM cultivar (ISAAA, 2018). In intention to release the first commercial genome edited plants, already since 2011, breeding companies and research institutions requested advice from national competent authorities, whether or how such plants are regulated and if they are subject to the same costly legal provisions that apply to GMOs. The planned commercial release by the company Cibus of a canola mutagenized for herbicide-tolerance with the use of ODM caused legal controversy in many countries and provoked controversial discussions on the regulation of genome edited products especially in the EU (for details see Sprink et al., 2016). The Court of Justice of the European Union (CJEU) ruled on directed mutagenesis (Case C-528/16) in July 2018 and provided regulatory clarity−with an unexpected outcome for European breeders and scientists working with genome editing. The ruling stated that in Europe the current legal GMO framework (2001/18/EC) applies to genome edited products without any exemptions which could only apply to organisms derived by random mutagenesis as they show a history of safe use before the law came into force. In contrast to the EU, Canada regulated the ODM canola just like any other crop plant without having a special view on the used technique reconsidering the novelty of its trait. The heterogeneity of the “regulatory mixture” could raise concerns and problems in the coming years when more and more products of genome editing will be released and not seen as a classical GMO (with all its requirements) in different crop producing and exporting countries. In the coming chapters, we will provide you with an overview of new developments in market-oriented genome editing applications and subsequently introduce the current regulatory approaches worldwide and demonstrate their differences.

**TABLE 1:** Genome editing related regulations in selected countries and determination whether or not SDN types lead to a GMO and respective regulations apply.

**Country**	**Laws/Regulations/Documents related to Genome Editing (release date)**	**CPB ratified?**	**InDels, substitutions**		**stable insertion of recombinant DNA based onSDN-3**
			**based on NHEJ; SDN-1**	**based on HDR using a template; SDN-2 and ODM**	

Argentina	Resolution No. 173/15 (2015)	no	consultation procedure; case-by-case decision		
			non-GMO		non-GMO* if not transgenic
Brazil	Normative Resolution No. 16 (2018)	accepted	previous consultation case-by-case decision		
			non-GMO		non-GMO* if not transgenic
Chile	Introduction of methodological procedure (2017)	no	previous consultation case-by-case decision		
			non-GMO		non-GMO* if not transgenic
Colombia	Resolution No. 00029299 (2019)	yes	previous consultation case-by-case decision non-GMO		non-GMO* if not transgenic
Paraguay	Resolution No. 565 (2019)	yes	case-by-case (unclear)		
Honduras	Agreement SENASA 008-2019 (2019)	yes	case-by-case non-GMO		non-GMO* if not transgenic
Guatemala El Salvador	Resolution UA 60-2019 + Annex: RT65.06.01:18 (2019)	accepted yes			
Israel	Seed regulations 5765– 2005 (Genetically Modified Plants and Organisms) (2005) after decision of the National Committee for Transgenic plants (2017)	no	non-GMO		transgenes: GMO cisgenes: non-GMO
Japan	Handling Procedures MHLW: Food Hygiene Handling Procedures for Food and Additives Derived from Genome Editing (2019); Notification by MOE: Handling of organisms obtained through the use of genome editing technology that do not fall under “genetically modified organisms” as defined in the Cartagena Act (2019)	yes	non-GMO, as long as no remnants of extracellularly processed DNA are integrated into the organisms genome		GMO (when extracellularly processed remnants of nucleic acid are integrated)
Australia	Gene Technology Amendment (Measures No. 1) to regulations (2019)	no	non-GMO	GMO	
United States	Statement from United States Agriculture Minister March 2018 and new SECURE Rules (2020) in§7 CFR part 340: Coordinated Framework for the Regulation of Biotechnology; Plant Protection Act; National Environmental Policy Act; Federal Insecticide, Fungicide, and Rodenticide Act (FIFRA)	no	USDA: case-by-case Differentiation between Food/Feed not regulated according to the process of genetic modification when no sequences of potential plant pests integrated FDA: voluntary process update/clarification under discussion		
Canada	Food and Drug Regulations (Division 28 of Part B) Directive 94-08 (CEPA) Seeds Act; Part V of the Seeds Regulations Directive 95-03, Guidelines for the Assessment of Novel Feeds: Plant Sources Health Canada’s Guidelines for the Safety Assessment of Novel Foods−Volume II	no	case-by-case (by novelty)		
China	“Administrative Rules for Safety of Agriculture GMOs”	approved	unclear new policies are under development		
Russian Federation	Decree No. 479 (2019)	no	currently unclear new policies are expected		
New Zealand	Hazardous Substances and New Organisms (HSNO) Act (1998) after court decision NZHC 1067 (2014)	yes	GMO		
EU	Directive 18/2001/EC (2001) after court decision in case C-528/16	yes	GMO		
India	*Draft Document on Genome Edited Organisms: Regulatory Framework and Guidelines for Risk Assessment (2020)*	yes	currently unclear (under discussion)		
Switzerland		yes	currently unclear (under discussion)		
Norway		yes	currently unclear (proposal under discussion)		
South Africa		no	currently unclear (under discussion)		

CPB, Cartagena Protocol of Biosafety. *Allele swaps unclear.

## New Developments in Market-Oriented Genome Editing Applications

Genome edited products are being developed worldwide. In a previous publication we have systematically identified applications of genome editing as a new tool for plant breeding (Modrzejewski et al., 2019). Progressing with this systematic search, we have identified market-oriented applications of genome editing techniques in crops and ornamentals between January 1996 and June 2019. A study has been considered as “market-oriented” when each of the following three criteria is fulfilled.

(1) A genome editing method (ZFN, Meganucleases, TALEN, CRISPR/Cas-technique or base editing) has been applied in a crop or ornamental plant.
(2) A trait has been edited that can be considered relevant for the crop market (e.g., biotic-/abiotic stress tolerance, herbicide resistance, etc.).
(3) It has been analyzed if the modified trait is distinct in the edited plant.

If the identified market-oriented studies will touch the market is not warranted, as the available data does not allow drawing conclusions on strategic decisions for further development and market releases. Some traits and plants are also models for basic research that cannot be distinguished from commercial perspectives by the given sources of information. The access to commercial data (e.g., from companies) is highly restricted and only available for very few selected approaches. In addition to scientific literature also databases like USDA-APHIS’ “Am I regulated?” have been evaluated and included in the survey.

**FIGURE 1:**
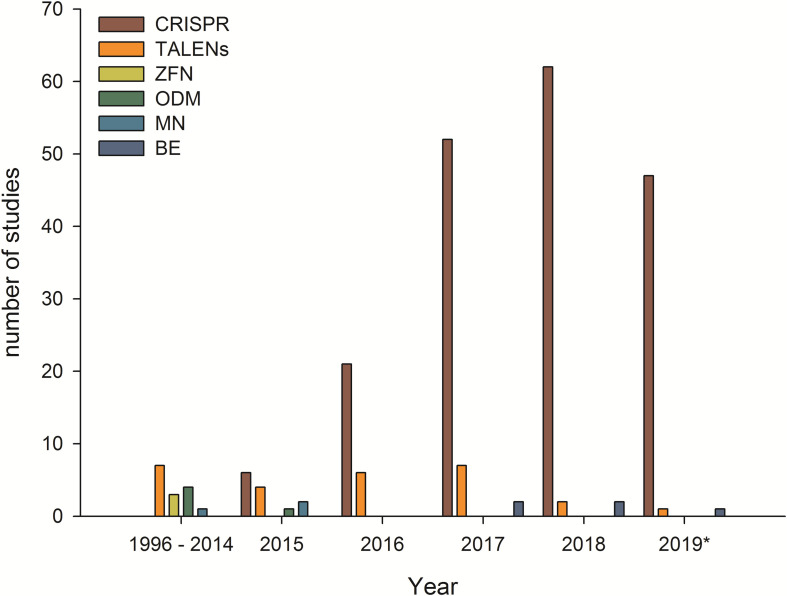
Number of studies with market-oriented genome editing applications in crop and ornamental plants in the timeframe January 1996 until July 2019. CRISPR/Cas9, Clustered Regularly Interspaced Short Palindromic Repeats/CRISPR associated protein 9; TALENs, Transcription activator-like effector nucleases; ZFN, Zinc-finger-nuclease; ODM, Oligonucleotide-directed Mutagenesis; MN, Meganuclease; BE, Base Editing.

**FIGURE 2:**
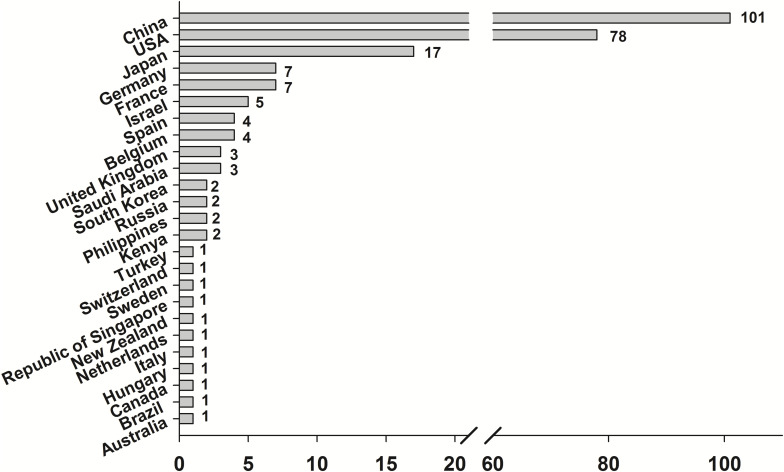
Number of market-oriented genome editing applications by countries in which the corresponding author is located. Multiple counts possible if the author has more than one affiliation or multiple corr. authors were named.

**FIGURE 3:**
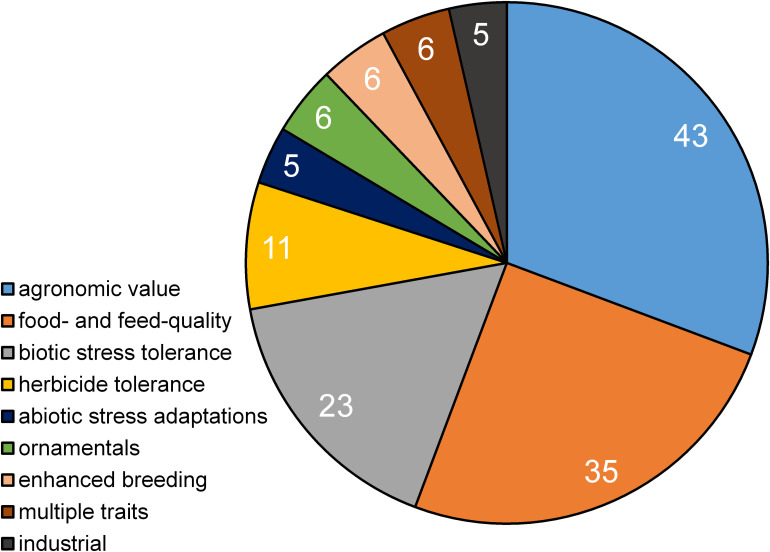
Distribution of genome editing traits by applications.

The following data are based on separate studies (one technique, one plant, and one trait). A publication may collate several studies as e.g., multiple traits have been addressed in a single plant using a single technique or a single trait has been edited in multiple plants. In total 217 publications comprising 231 market-oriented studies have been identified. Most of the studies have been performed using a CRISPR/Cas-System (Figure 1). The corresponding authors emerged from 25 different countries. Most of them have been based in China (101) followed by the United States (78) and Japan (17) (Figure 2). Market-oriented applications could be identified in 41 crop plants and ornamentals. The majority of the market-oriented applications have been performed in rice (81) and tomato (26) followed by the main staple crops maize (25), wheat (14), potato (14), and soy (12). Besides these main crops, there are studies in peanut, kiwi, lettuce, lemon, poppy, salvia, cacao, banana, manioc and sugar cane. In total 140 different applications were identified A detailed list of traits modified by genome editing can be found in Supplementary Table 1. Most of the traits can be summarized as agronomic traits (43) followed by food and feed quality (35) and biotic stress tolerance (23). All traits are shown in Figure 3.

## The Global Regulatory Status of Genome Editing in Plants in 2020

Although numerous countries are working on the development of market-oriented crops for several years, only a handful of countries clarified their opinion toward genome editing between 2014 and 2016 (see Sprink et al., 2016; Ishii and Araki, 2017). However, in the last three years more and more countries adopted legislation, amended their current regulations or clarified the interpretation of their legislations with regards to genome editing and its products. Especially, countries with product-oriented regulatory concepts and long lasting GMO cultivation in the field like Canada or the United States did not change their regulatory system at all and genome edited plants passed without specific regulatory burdens. New Zealand developed a new frame work already back in 2014 but had to stick with its old GMO regulations after a high court decision just like Europe. Nevertheless, controversial discussions are ongoing both in the EU and in New Zealand, as more and more (partnering) countries promote genome editing and products thereof such as Israel and more recently Japan and Australia which stated not to regulate plants derived by some types of genome editing.

The first countries that released advices, opinions or regulations on genome editing are located on the American continent namely Argentina, Chile, the United States, and Canada. Furthermore, Brazil, Colombia and Paraguay enacted normative resolutions on genome editing after Argentina and Chile had released their resolutions. Some of these legislations unambiguously name the techniques of genome editing, which do or do not lead to a GMO. Hence, local breeders and producers gain clarity beforehand (see Table 2).

**TABLE 2:** Regulatory status of genome edited products in Latin America ordered chronologically, source: ANPROS – modified and appended.

	**Argentina**	**Chile**	**Brazil**	**Colombia**	**Paraguay**	**Honduras (together**	**with) Guatemala and El Salvador**
Legal Basis	Resolution 173-2015 (Ministerio de Agricultura, Ganadería y Pesca−Argentina, Argentina.gob.ar, 2015)	Consultation Procedure	Resolution 16-2018 (CTNBio, 2018)	Resolution 00029299-2018 (Instituto Colombia Agropecuarioa (ICA), 2018)	Resolution 565-2019 (Ministerio de agricultura y ganaderia, 2019)	Agreement SENASA 008-2019 (SAG-SENASA, 2019)	Resolution UA 60-2019 (Customs union of El Salvador Guatemala and Honduras, 2019) and Annex: RT 65.06.01:18
Release Year	2015	2017	2018	2018	2019	2019	2019
NPBT listed	no	no	Yes	no	yes	no	no
Definition of genome editing	missing	missing	In Annex I: Oligonucleotide/Site directed mutagenesis	missing	New Breeding Techniques: CR(Y/I)SPR, TALEN, and others	New Techniques of genetic improvement (precision biotechnology)	Organisms obtained through the application of modern biotechnology
Assessment **(GMO or Not?)**	60 days	20 days	90 days + extended to 120 days	60 days	No information	45 days	90 days
Communication	Not public	Officially published	Officially published	Officially published	−	−	−

In many other countries the legal status of genome editing is not decided yet or still under discussion. Examples are Norway or Switzerland but also in Russia and India the regulation of genome-editing is still in a debate with open outcome. Several African countries are currently debating as well. To date, South Africa and Sudan grow genetically modified crops while South Africa has already begun discussing genome editing and related regulation. Recently, Burkina Faso, Nigeria and Ghana started cropping GM-plants and Uganda still debates the establishment of GMO legislation without explicitly naming genome editing. The recent declaration of the Africa Biennial Biosciences Communication (ABBC) Symposium stated that regulatory frameworks should facilitate the access to genome editing and awareness on genome editing should be created among African policy and decision-makers (ABBC, 2019). Lately, also India has just released a draft document on Genome Edited Organisms in which they suggested a tiered risk approach for the regulation of genome-editing products. Their draft is open for discussion with stakeholders.

In the following, we shed light on genome editing related regulations or intended law amendments for prominent countries based on available/accessible information in the recent time period up to 2020. We sorted the countries on basis of their progress (decided, undecided yet and in discussion) to integrate genome editing into their regulations and furthermore have condensed the available information in Table 1.

### Latin American States

Since 2015, Latin American countries established explicit regulations for genome editing. Argentina released a resolution in 2015, Chile followed in 2017. In 2018 and 2019, Brazil, Colombia and Paraguay released their resolutions (see Table 2) and the Central American countries Guatemala, Honduras and El Salvador introduced a common biotech policy. Furthermore, Uruguay and the Dominican Republic positioned within the WTO for a policy regarding genome editing based on scientific consensus. The policies in Latin America extended the existing regulatory GMO frameworks with resolutions to clarify the legal status of genome edited organisms. Common for all policies is that an interested party consults or notifies a respective agency in a mandatory or voluntary procedure e.g., CONABIA in Argentina (Table 2). Within a given time (20–120 days) the agencies determine case-by-case whether a plant product falls into the country’s GMO/LMO definition and whether the obligations for GMO apply. Provided that there are no residues of foreign recombinant DNA detectable in the plant, InDel or base substitutions in the plants genome carried out by SDN-1, SDN-2, or ODM do not result in a GMO. Evidence of absence of a transgene (residue) must be provided to allow for exemption from consideration as GMO (Whelan and Lema, 2015). Brazil and Paraguay especially name the techniques that do not lead to a GMO. Since SDN-3 introduces in most cases recombinant DNA sequences of foreign origin into the plants genome, it leads to a GMO categorization. An exceptional SDN-3 case is the complete replacement of an allele with another (allele swap). In this case, the GMO status is seemingly unclear. Although not represented in scientific data (Modrzejewski et al., 2019), Argentina and other South American countries are among the top growers of genome edited crops in field trials for research purpose as well as for propagation mainly for United States based companies (Calyxt Inc, 2016). As all field trials performed in Argentina -and elsewhere in Latin America- are confidential, no official numbers are published concerning the number of neither events nor the plot sizes. The only available resource is the voluntary disclosures by companies performing such trials.

### United States of America

**FIGURE 4:**
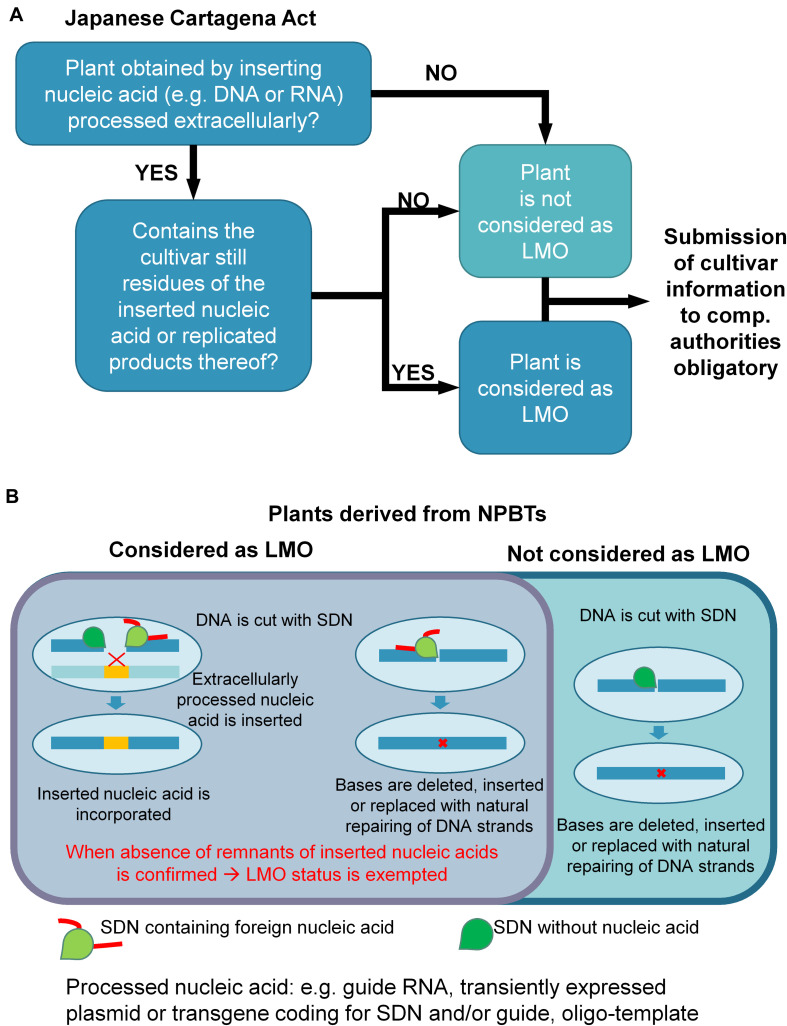
Updated scheme of the §7CFR Parts 340 (SECURE Rule) with a decision tree for a developer/breeder/interested party to determine whether §7 CFR Part 340 applies to a new genome edited plant or whether it is excluded; created on the basis of the published SECURE rule.

The United States regulatory system has not been changed with the emergence of genome editing and products thereof. In fact, genome editing was incorporated in a lasting discussion of renewing the biotech regulation. As in 2015, the Administration of President Obama issued a memorandum directing Environmental Protection Agency (EPA), Food and Drug Administration (FDA), and the United States Department of Agriculture (USDA) to clarify the roles and responsibilities of these agencies regulating biotechnology products under the Coordinated Framework for the Regulation of Biotechnology (Coordinated Framework). Plans were published to modernize the Coordinated Framework for the regulation of biotechnology (EPA, 2017), providing information about the types of biotechnology product areas regulated by each competent authority (i.e., EPA, FDA, or USDA). The US Secretary of Agriculture reconfirmed in March 2018 that USDA’s Animal and Plant Health Inspection Service (APHIS), which is the primary regulatory authority for plant products in the United States, does not regulate or has any plans to regulate plants that otherwise could have been developed through traditional breeding techniques under §7 CFR part 340 which is the operative statue governing regulation of genetic engineering within APHIS (USDA APHIS, 2020). Also in FDA (2018), the FDA committed in context of its Plant and Animal Biotechnology Innovation Action Plan to pursue advances in policy priorities in order to establish a science-and-risk-based approach for product developers and to remove barriers for future innovation in plant and animal biotechnology. Under the Administration of President Trump, at The White House (2019), an Executive Order on Modernizing the Regulatory Framework for Agriculture Biotechnology Products described reforms to promote agricultural innovation and streamline current regulations for biotechnology and emerging technologies. In line with this order APHIS proposed a revision of the §7 CFR part 340 (USDA APHIS, 2019). The new proposal was a product of several feedback phases with stakeholders and asked for public comment on the proposed revisions. In USDA APHIS (2019), the proposal was implemented in the new SECURE rule as a comprehensive revision of APHIS’ biotechnology regulations and will be fully implemented by 2021. The revised framework is meant to provide clear, predictable and efficient regulatory pathways for applicants, when the plant products are unlikely to pose a plant pest risk (Figure 4). In effect, the new rule exempts categories of products developed through genome editing under most conditions from obligations under §7 CFR part 340 when changes in the plant *product’s* genome are:

(1) deletion(s) of any size;
(2) targeted substitutions of a single base pair; or
(3) solely introductions from sequences derived from the plant’s natural gene pool or edits from sequences which are known to correspond in the plants natural gene pool.

Although not explicitly named, the classification resembles mostly the classification by SDN-1/-2/-3. In addition, APHIS may exempt product depending on the individual case (Figure 4). Furthermore, when a new plant contains plant-trait-mechanism of action previously evaluated by APHIS e.g., in a transgenic plant, and found there already to be unlikely to pose a plant pest risk, it is also exempt from regulation. This will facilitate applications with similar combinations of plant-trait-mechanisms of action and corresponds to the usual procedure for genome edited plants which are not *per se* considered by APHIS to be plant pests and thus not as regulated article, provided that no plant pest derived DNA-sequences have been integrated or used as template.

In a new exemptions and confirmation process applicants may request confirmation that their products are exempted and APHIS will provide a written confirmation within 120 days. This replaces the previously offered voluntary consultation process “Am I regulated according to 7 CFR parts 340?” (AIR) which allowed interested parties to determine the regulatory status according to plant pest character prior marketing in the United States. AIR was discontinued on the 16th of June 2020. Of the 86 inquiries listed in AIR since 2010, USDA APHIS exempted more than 35 using genome editing. These stay exempted also in the new guidelines. Notably, besides big agricultural companies numerous small and medium enterprises and academic institutions are among the inquirers. These received a published decision typically within 3−6 months. In almost all responses from APHIS, one advises the interested party to consult also with FDA and EPA but both agencies do not offer a comparable service to APHIS so far. The FDA has its responsibility in food additives. Therefore, the FDA evaluates the safety of plant derived foods and feed products through a consultation procedure. The consultation procedure is not based or started by using a specific breeding procedure *per se*. FDA firms up on a product based regulation by comparing substantial equivalences of a novel food product to a known comparator. This regulation is linked to the properties of the novel food (e.g., purpose, composition, structure and use), only if corresponding references between novel food and comparator are missing e.g., changed allergens, FDA requires an approval procedure. The evaluation is completed when FDA has no further questions to the interested party (CAST, 2018). EPA’s responsibilities are related to products generating pesticides (e.g., Bt-Toxins) or to food containing pesticide residues. As many genome edited products will not produce pesticides *per se* it is expected that EPA will only play a minor role in the evaluation and release of most genome edited crops in the United States.

In the meantime, United States farmers are growing the first genome edited plants. The ODM based genome edited canola cultivar of Cibus, which was controversially discussed in EU, is marketed since several years in the United States without any formal approval and AIR inquiry. Since fall 2018, a soybean with modified oil composition was harvested at small scale as the first TALEN-based genome edited crop. The cultivation area increased to approximately 17 000 hectares in 2019. The company Calyxt which developed the soybean is marketing it as an identity preserved product by contracting with farmers and purchasers. Calyxt has developed the new soy cultivar, distributed the seeds to contract farmers and commercializes the derived product High Oleic Soybean Oil as a high-quality food ingredient (Calyxt Inc, 2019). By 2020, the acreage will increase to approximately 40 000 ha. Field trials of these new soybean cultivars were conducted already during Calyxt Inc (2015) in the United States and Argentina. With Yield10, another company is planning to conduct field trials with the first CRISPR/Cas edited canola as a consequence to APHIS’ approval in Yield10 Bioscience Inc (2020). Further genome edited plant products are in the pipelines of SME biotech companies as well as international plant breeders. So, more products will follow in the coming months presumably without severe regulatory hurdles in the United States as the first crops like soybean and canola have successfully undergone this procedure without major problems (FDA, 2019).

### Canada

Just like in the United States, Canada’s regulatory system has not been changed with the emergence of genome editing, but due to its product-oriented policy the system is flexible and able to cope with all plants, irrespective of their breeding method (Smyth, 2017). All plant products, whether obtained through biotechnology (e.g., transgenesis or genome editing) or conventional breeding including classical mutagenesis, are subject to the same oversight in the regulatory framework for plants with novel traits (PNTs). The Canadian legislation is based on novelty of product characteristics what differs from other regulatory frameworks. All products are considered case-by-case based determination on novelty. Plant products classified as PNT are subject to extended oversight and are tested for allergenicity, toxicity and impact on non-target organisms. In the product-based legislation there is no clear definition of novelty, but a rule of thumb of about 20% difference in the respective trait(s) to a reference product has been established (Smyth, 2017). The Canadian Food Inspection Agency (CFIA) offers guidance to determine novelty and when to notify the agency. Canada already approved herbicide tolerant Canola developed by Cibus in 2017 but the corresponding entry in the registered variety database does not exist anymore. The reasons for that are unclear.

### Israel

In March 2017 Israel reconfirmed the statement from 2016 that plants modified by genome editing are not subjected to the Seed Act (Genetically Modified Plants and Organisms) from 2005 and will not be considered as GMO. The National Committee for Transgenic Plants published its decision that genome edited plants do not fall under the regulation when only small deletions or sequence edits occurred (USDA FAS, 2018a). Interested parties introducing new cultivars to Israel need to demonstrate that no foreign DNA was incorporated in the organism’s genome. Recently, Israel’s Ministry of Agriculture announced the plans to invest approximately 17 Million US-Dollar to establish a national genome editing center (Minister of Agriculture and Rural Development, 2019). Israel strongly promotes research and development of new and innovative agricultural products in the plant and livestock fields.

### Japan

**FIGURE 5:**
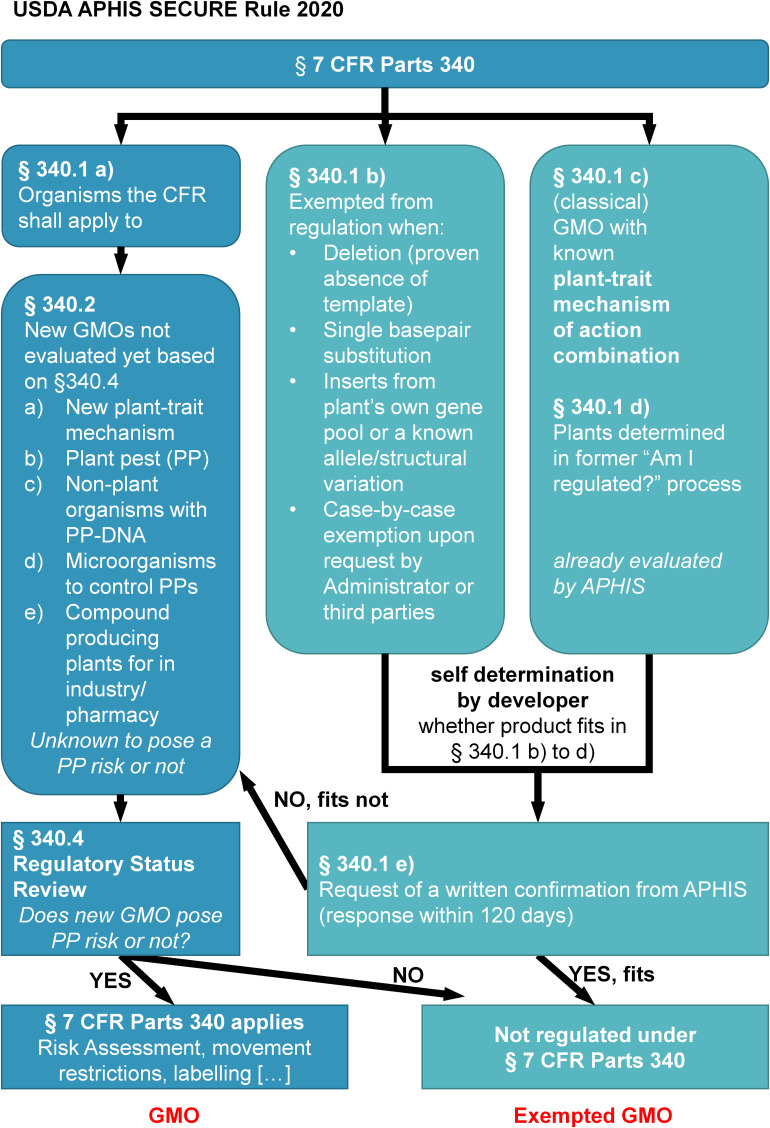
Decision tree of Japanese regulation based on the practical guide released by the Japanese Ministry of the Environment in 2018 (modified and appended based on decision guidance, MoE Japan (2018)). **(A)** Decision tree to test if plants fall into regulation of Japanese Cartagena Act and the regulations stipulated therein. **(B)** The technique (e.g., type of SDN) and outcome is important to decide whether or not plants are seen as living modified organism (LMO) according to Japanese Cartagena Act.

The Japanese cabinet decided in Japanese Cabinet Office (2018) in respect of its integrated innovation strategy, that the handling, cultivation and release of genome edited organisms should be clarified under the Cartagena Act and Food Sanitation act by the end of March 2019. Thereupon, an expert panel of the Japanese Ministry of the Environment (MOE) suggested that organisms derived by SDN-1 should not be regulated (Igarashi and Hatta, 2018; USDA FAS, 2018b). In MoE Japan (2018), released a practical guide addressed to interested parties to resolve which genome editing technologies result in LMO according to Cartagena Act and which information should be provided to the respective ministries/authorities. In March 2019, the MOE clarified its genome-editing policy: Organisms modified by insertion of *extracellularly processed* nucleic acids generally result in LMOs and the obligations of the Japanese Cartagena Act apply. Exemptions can be made when the absence (e.g., by outcrossing) of inserted nucleic acids or its replicated products in the genome were confirmed (USDA FAS, 2019a) and only the genome-edit(s) remain in the genome. Accordingly, organisms derived by SDN-1 are exempted from regulation, when no foreign sequences (e.g., coding for the SDN) remain in the genome (see Figure 5). For SDN-2 and ODM where extracellularly processed nucleic acid sequences pose as template for HDR, absence of these must be demonstrated. No information is given, how the absence should be proven. Exceptions are therefore handled on a case-by-case basis. SDN-3 applications are generally regarded as LMO although it is not clarified, how allele swaps will be regulated. Irrespective of the applied method, the competent authority needs to be informed in every case on the edited sequences in the organism.

For food derived from genome edited organisms, the regulations of the Ministry of Health, Labour and Welfare (MHLW) apply. In MHLW Japan (2019) the Councilor for Environmental Health and Food Safety released handling procedures for the marketing of food and feed products and additives, entirely or partially derived from genome editing or from crossbred genome edited progeny. Before placing on the market, producers need to consult the MHLW about the regulatory status of the respective product. The MHWL determines case-by-case whether a specific safety assessment is required (as for GM food) or a notification is sufficient. A notification requires information on the editing technique, the genes targeted for modification, year and month of marketing and other details from developers or in case of imported product from importers. After its publication by the MHLW marketing will be enabled; if food is processed from notified genome edited products a separate notification is not required. For food, SDN-1 and SDN-2 derived products harboring genomic substitutions or indels of one to several bases are considered as similar to conventional products and a notification is in most cases sufficient; while for food derived from SDN-3 containing foreign genes a safety assessment is mandatory. It remains unclear which method will be accepted to prove the absence of transgenes or extracellularly processed nucleic acid sequences in the final product. Such a decision is expected to be made on a case-by-case basis. One of the beneficiaries of the new regulation is the Japan based company SANATECH-Seeds, which developed a genome edited high GABA tomato which test market release is planned for 2021.

### Australia

In 2016, the Australian government initiated the third review of their National Gene Technology Scheme to clarify the scope of regulation in light of ongoing technical progress. The initiative involved national and international stakeholders. The final report was released in Australian Government - The Department of Health (2018). In order to facilitate the flexibility of the regulatory scheme, several recommendations proposed a reorganization of the legislation and the establishment of a risk tiering. It was proposed to ensure a regulatory level proportionate to risk and to avoid over-regulation or under-regulation, respectively. Based on the identification of new risks or the history of safe use, allocation of organisms between categories should be ensured with appropriate flexibility. Furthermore, the existing legal process-based trigger and the subsequent risk-assessment were maintained in the review (Australian Government - The Department of Health, 2018). Instead of reorganizing the entire legislation as proposed, the Australian government officially published a first set of updated amendments of the Gene Technology Scheme in April 2019. Genome editing is defined as gene-technology in Australia. Organisms modified with SDN-1 are exempted from regulation as a nucleic acid template was not added. Conversely, this means that ODM, SDN-2, and also SDN-3 are regulated as GMO (Mallapaty, 2019; Thygesen, 2019). The majority of amendments in Australian GMO regulation were implemented on Australian Federal Executive Council (2019). Furthermore, the Australian federal state Tasmania campaigns for a strict regulation of SDN-1 modified organisms as GMOs within its shorelines (Maloney, 2019). For products from genome editing designated for human consumption, different rules will apply and are still under discussion (see New Zealand).

### New Zealand

Already in Environmental Protection Authority (2013), with increasing awareness of genome editing in plants New Zealand’s environmental protection authority decided about their status as being a non-GMO Plants genome edited by SDN-1 types were considered closely related to plants treated by chemical mutagenesis and were exempted from New Zealand’s GMO regulation in the Hazardous Substances and New Organisms (HSNO) act (Ministry for the Environment New Zealand, 1998). This interpretation was overturned by a High Court decision in Kershen (2015). Hereupon, the act was updated clarifying that all mutagenesis techniques established after 1996 lead to GMOs. Since then, genome edited plants are regulated as GMOs in New Zealand and the respective biosafety regulations of the HSNO act apply (Kershen, 2015).

For developing food and feed standards, New Zealand and Australia jointly operate the statutory authority FSANZ (Food Standards Australia New Zealand). Their standards are published (“Food Standards Code”) and apply to food produced for sale in, or imported into Australia and New Zealand. In FSANZ (2018a) started a stakeholder consultation to determine whether such imports need a pre-market assessment and approval as it is established for classical GMOs. A preliminary report was released quoting and summarizing the responses to key questions (FSANZ, 2018b). Many stakeholders pointed to differences in definitions of genome editing between the laws in New Zealand and Australia and the common Food Standards Code. Furthermore, stakeholders consider harmonization of regulatory approaches to genome editing, both domestically and internationally as the way to facilitate trade and certainty while providing the agricultural sector and consumers with access to innovative products (FSANZ, 2018b). The final report of the consultations is still pending but gained importance due to the deregulation of SDN-1 based genome editing in Australia in 2019. Additionally in 2019, New Zealand’s Royal Society released a critical opinion about the current situation of genome editing in New Zealand and proposed options to change current legal obligations proportionate to risk and in accordance to Australian proceedings in genome editing regulations (Royal Society/Te Aparangi (ed.), 2019).

### European Union

With its emergence, the legal situation of genome editing has been discussed lively in Europe (Eriksson, 2018). In July 2018 the European Court of Justice (CJEU) ruled that products resulting from targeted mutagenesis methods are regulated under the full provisions of the Directive 2001/18/EC for the deliberate release of GMOs. Products derived by undirected mutagenesis (chemical- or radiation-induced) are legally considered GMOs as well, but stay exempted from further obligations according to Annex 1B Directive 2001/18 and due to their long safety record as followed from recital 17 (Court of Justice of the European Union, 2018). Due to the ruling previous legal interpretations or decisions of EU competent authorities became obsolete and needed to be retracted. However, new challenges do arise from this decision: The EU is now challenged to enforce the judgment and the Member States are obliged to monitor compliance with regards to (unauthorized) genome edited plants and products derived thereof. Their identification and differentiation is currently hardly possible (Grohmann et al., 2019). The European Network of GMO Laboratories (ENGL) emphasized the difficulties and technical limits in identifying genome edited plants. The enforcement of the current European GMO legislation thus is challenging (ENGL, 2019). Besides the CJEU judgment clarifying the interpretation of the current GMO-legislation in respect to genome editing, the Commission did not yet provide any plans for an update of the Directive. On request of the European Council the new elected European Commission initiated a study to be finished in April 2021 regarding the status of “novel genomic techniques” after the ruling and the implementation (Council of the European Union, 2019). Hence, any further initiative by the Commission to update the legislation is not expected before 2021.

Proceeding from the ruling of the CJEU the French high court recently ruled that in France besides genome editing also organisms obtained through (classical) “*in vitro* mutagenesis” are subjects of the GMO regulation. The ruling forced the French government to update the French Environment Code (as the national implementation of the European GMO directive) within 6 months. In this respect, the French government recently released a draft (European Commission, 2020): France intends to prohibit the cultivation or trading of such varieties (in France) unless there is an approval according to the GMO regulations. This solo could cause trade problems, as some varieties thus will be banned in France but are unrestrict the other Member States. The consistency of this French interpretation of the CJEU ruling with regards to the EC directives is thus yet under discussion (Bartsch et al., 2020).

### China

China massively invests in genome editing research and is a leading country regarding in genome-editing publications (Cohen and Desai, 2019). The country has set a strong focus on improvement of market-oriented traits in crops (see below) (Cohen, 2019). A growing number of published research with genome edited crops grown in field trials on Chinese territory in the recent years, e.g., in the staple-crops maize (Chen et al., 2018) and rice (Tang et al., 2017; Miao et al., 2018; Songmei et al., 2019; Yang et al., 2019), demonstrate the ease to test genome edited crops under field conditions in China. Moreover this proves the willingness of the Chinese government to promote genome editing. Up to now, China has not released any genome editing related legislation or technical comment. Discussion on risk analysis of genome editing derived products has been initiated in China since 2015 and a working group within the National Biosafety Committee (NBC) was established in September 2016 to provide technical assistance on how to regulate new techniques including genome editing in China. Formal regulations have not been issued yet (Gao et al., 2018). The Chinese government closely monitors foreign policies on genome editing (USDA FAS, 2019b). The Ministry of Agriculture and Rural Affairs (MARA) is paying close attention to the activities of the U.S. FDA and EPA and other countries’ regulatory agencies concerning how those organizations regulate genome editing (USDA FAS, 2019b). Due to China’s own strong investment in genome editing, one expects a release of a genome editing-friendly policy by the Chinese Government in the next years. Furthermore, China has already paved the way for releasing genome edited varieties by acquiring Syngenta through the state owned company ChemChina. One of Chinas leading CRISPR/Cas-researchers Caixa Gao stated recently that it would only take them 6 month to bring a variety (e.g., aromatic elite rice varieties) from the lab to the farm (Cohen, 2019).

### Russian Federation

Since 2016 the Russian Federation law prohibits the cultivation of genetically modified plants and the breeding of genetically modified animals on the territory of the Russian Federation, except for the cultivation and breeding of plants and animals required for scientific or research purpose. In June 2017 the Government of the Russian Federation issued Resolution No. 770 which amends Russia’s regulatory framework for the registration of GMOs and products derived thereof or containing such organisms in order to continue the ban on cultivation and breeding of GMOs (Federal Law No. 358 of July 3, 2016) (USDA FAS, 2018c). Regarding the development of genome editing in the Russian federation, little information is actually available. Most domestic research in agricultural biotechnology is limited to biological means of plant protection, growth stimulators, and microbiological fertilizer (USDA FAS, 2018c). Whether the Russian Federation regards a genome edited organism as being a GMO and is prohibited for market release, is unknown. Furthermore, in order to execute a potential ban, it is unknown how Russian authorities will monitor compliance. The apparent anti-GMO attitude of the Russian government changed in Russian Government (2019), when the Russian Ministry of Education and Science issued the decree No. 479 to reduce the deficits in the Russian biotechnology and to address the development in genetic technologies including gene editing. With the decree a billion dollar research program is initiated (Dobrovidova, 2019). Besides animal and medicinal biotechnology, the financing will support the improvement of genome editing in essential plants for the Russian agricultural production: barley, wheat, potatoes, sugar beet and fast-growing tree lines. The decree defines plant products derived by “*some types”* of genome editing as being equivalent to those derived by conventional plant breeding. Moreover, hindering regulations were named and recommendations for improvements provided. Therefore, an update of the Russian regulations that promotes genome editing is expected in the next years. The first company to take advantage of this new opportunity is Doka Gene Technologies Ltd., which in cooperation with the Moscow State University intends to apply genome editing to create a Potato Virus Y-resistant potato (Makhotenko et al., 2019).

### India

In Indian Ministry of Science and Technology (2020) the Indian Department of Biotechnology drafted genome editing guidelines. The guidelines propose a tiered regulatory approval process based on categorization in regulatory groups depending on genome editing type. Group 1 combines plants whose genomes harbor one or a few base pair edits or deletions based on SDN-1 or ODM, whereas in Group 2 are plants which harbor a few or several base pair edits based on SDN-2 using a template. The distinction between *a few* and *several* is not conclusive in the draft. Risk assessment in Group 1 and Group 2 should require confirming the targeted edit and to rule out biologically significant off-targets as well as testing for the efficacy of the traits, for their equivalence to reference varieties except the edited trait, generally on a case-by-case basis. The third group is for plants with large DNA changes and insertion of foreign DNA. In that case, the same stringent risk assessment as for classic transgenic plants applies.

### Switzerland

In Generalsekretariat UVEK (2018) the Swiss Federal Council released its plans to modify the current gene technology regulations in order to adapt them to the latest developments in genome editing. According to a study from the Federal Department of the Environment, Transport, Energy and Communications (DETEC) and Federal Department of Economic Affairs, Education and Research (EAER), the current Swiss GMO regulation from 2004 is inadequate to cope with plants derived from genome editing and whether genome edited plants should be considered as genetically modified organisms or not (Transkript, 2018). Switzerland has a moratorium on GMO cultivation until the end of 2021 and it is unclear if the moratorium encompasses also plants derived from genome editing. Any new regulation is planned to include a categorization of products and technologies into different risk classes. A first outcome of the debate was expected by the end of Hardegger (2019), but an official statement is pending. It has to be noted that Switzerland is located inside the EU territory and regulations are affected by transboundary trade issues.

### Norway

Currently, the Norwegian GMO authorization process is entangled with the European authorization procedure (Eriksson et al., 2017). In Norwegian Biotechnology Advisory Board (2018), the Norwegian Biotechnology Advisory Board proposed a re-evaluation of the Norwegian regulatory framework for GMO. Currently, classical GMOs and products derived by genome editing are categorized into four risk tiers based on differences in genetic modification technology, organism, potential of invasiveness and social parameters (Bioteknologirådet, 2018). Relevant criteria are stability (stable/permanent change) and thus heritability of a genetic modification, whether the change could have been induced using conventional breeding techniques, and whether the change crosses species boundaries. For an organism or product categorized in the lowest level, a notification of competent authorities (and their response) may be sufficient. At higher categories, organisms would require approval before release, and may be subject to more stringent risk management requirements. An official statement by the Norwegian government how NPBT derived plants will be regulated, is still missing. Norway is an associated country to the EU with regards to trade and travel. Hence uncoordinated policies may cause unprecedented issues.

## Developments in Global Organizations

### OECD

The Organization for Economic Co-operation and Development (OECD) recognizes increasing impact of emerging new breeding technologies such as genome editing on global economies. For that reason, the OECD Conference on Genome Editing: Applications in Agriculture was held in June 2018 bringing together relevant stakeholders like scientific experts, decision makers, company representatives and others from more than 35 countries (*OECD Review of Fisheries: Policies and Summary Statistics 2017*, OECD, 2017; Friedrichs et al., 2019). Participants discussed that regulatory approaches for genome editing should be determined to achieve policy objectives considering both, precaution and innovation through better communication between all stakeholders (Friedrichs et al., 2019). Furthermore, it is important that the different legal systems understand their respective regulatory and policy approaches to genome editing. To minimize difficulties arising through different regulatory approaches a common understanding is obligatory (Friedrichs et al., 2019).

### WTO: Committee on Sanitary and Phytosanitary Measures

In November 2018, the delegations of Australia, Argentina, Brazil, Canada, the Dominican Republic, Guatemala, Honduras, Paraguay, the United States of America, and Uruguay signed the international statement on agricultural applications of precision biotechnology in the WTO Committee on Sanitary and Phytosanitary Measures (CSPM). The delegations agreed to engage for the exploration of science based opportunities for regulatory frameworks and the avoidance of trade barriers for products derived from genome editing (Commitee on Sanitary and Phytosanitary Measures, 2018). In their declaration the states affirmed that cultivars derived from genome editing should be regulated similar to conventional cultivars due to their high similarity. Deregulation of genome editing techniques offers new opportunities for SMEs and national research institutions. Thus, a harmonization at national and international level should be ensured to exploit the full potential of genome editing. Furthermore, within the CSPM the United States with support from Argentina and Paraguay raised specific trade concerns (STC 452) about restrictions from the European Union resulting from the implementation of the CJEU Ruling in Commitee on Sanitary and Phytosanitary Measures (2019). The implementation would lead to unjustified barriers to trade in products of genome editing. It stifles the agricultural research and innovation necessary to prevent hunger and malnutrition in the coming decades, while ensuring environmental sustainability of agricultural activities. Without any changes in European legislation the issue stays unresolved.

## Conclusion

In this review, we could show that genome editing and related regulations engender growing interest in recent years in all regions of the world. An enormous increase of studies performing genome editing in crops has been recognized. Especially, the CRISPR/Cas and related systems (e.g., base editors) have been used lately for almost all studies and a rising number of market-oriented traits have been addressed by genome editing. We have identified that not only agriculture relevant traits but especially product quality traits (e.g., better digestibility or reduced allergen content) are being worked on. Even though, a multitude of traits have been edited, only few genome edited plants (a soybean cultivar by Calyxt and canola by Cibus) have reached the market so far, but more crops are in the pipelines of various companies and may soon appear on the market. A reason why not many more of the identified market-oriented applications reached the market in the last decade, might be that products of genome editing are regulated differently in different countries. Their regulation is triggered either by the product itself or on the methods used (process). The global “regulatory mixture” sets high hurdles for the global release but also for import (and export) of genome edited plants, so the first genome edited crops released so far, were marketed in countries with a genome editing friendly policy. Moreover, we have identified that most countries, which are active in developing market-oriented traits have such a friendly policy (e.g., United States and Japan). Interestingly, the most active country in genome editing research, China, has not released any legal documents so far, the reasons for this are unclear, but it is possible that China wants to have a product in hand before releasing regulations. Europe which has a strict policy toward genome editing is among the leaders in genome editing research (Modrzejewski et al., 2019) but is lagging behind when it comes to market-oriented trait development. This field is currently led by China and the United States.

The regulation of breeding technologies largely differs between countries and depends in most cases on whether modifications appeared as *natural* mutations, *untargeted* due to radiation-based or chemical mutagenesis or *targeted* by the use of transgenesis or genome editing technologies. Comparing the regulation of genome editing and its products we have identified different approaches. None of the mentioned countries followed the approach of a complete reorganization of their regulatory systems. Instead, several countries kept their current framework (e.g., Europe, New Zealand, and Canada), which is leading to a comparable outcome of regulation as GMOs in the respective countries. Another approach is to amend existing regulations or to release resolutions (e.g., South American countries, Japan). These approaches have in common that they make use of a case-by-case evaluation and a tiered approach, based mainly on the SDN1-3 categories (Table 1). Others like Australia and more recently United States streamlined certain processes in order to improve legal clarity for genome edited plants. Most South American countries with updated policies furthermore require the applicant to pre-consult the competent authority before officially starting an approval of a genome edited crop for that country’s market. The assessment of such a pre-consultation request is conducted within a given time frame and helps the applicant to predict the duration and cost of regulatory scrutiny before beginning an official market approval. Moreover, pre-consultation reduces the risk of non-approval, provided that all necessary information is available to the authorities.

When legislations are focusing on the product, the type of SDN and presence of foreign sequences are generally in most countries the most important factors for the legal classification of a genome edited plant. Plants with genome edits like deletions (and small insertions) of one or several bases which explicitly lack any introduced foreign sequences, are exempted from GMO status. The absence of potential transgenes must be ensured, so that in derived null-segregants the genome edit(s) are kept while sequences, e.g., of the SDN are absent. Consequently, e.g., in Japan or the United States for SDN-1 edited plants it is sufficient to notify the competent authority that the plant is free of transgenes to exclude them from GMO legislation. Which proof is sufficient, that foreign DNA has been removed will be decided on a case-by-case basis as no legislation provides precise guidance for this issue? The detection of genome insertions of foreign DNA (transgenes) is generally straightforward whereas genome edits by SDN-1 are not distinguishable from natural occurring mutations or classically bred plants (Grohmann et al., 2019). Thus, a legal basis to differentiate “artificial” from “natural” is not given and could explain why most product-based legislations do not regulate SDN-1 based edits. The situation is different for larger insertions and editing by SDN-2 or SDN-3, where mostly externally modified sequences were used. These are usually detectable and make the plant distinguishable to classically bred plant. In the process-based legislations of European Union and New Zealand, on the other hand, the process of genome editing itself and not the product is already decisive for stricter regulation, so the SDN type is -by now- irrelevant for legal classification.

Generally, the outcome of case-by-case approvals is still difficult to predict, but as more and more products will enter the markets, the predictability of whether or not new genome-editing developments will have a chance of obtaining marketing approval outside strict GMO guidelines -as in Europe and New Zealand- will increase. Where the global journey of genome editing regulation is heading is not clear yet. As all regulations differ at least in details and some countries follow a complete different path compared to the currently released legislations a global harmonization of genome editing regulations is in far distance. Even more complex regulatory systems based on tiered approaches could appear in the near future as proposed by Norway, Switzerland and India. Challenges associated with genome editing might be on the agenda of undecided countries earlier than expected as main questions concerning detection and identification as well as free global trade remains unresolved. It can be only speculated if the expected study from the European commission in April 2021 will clarify some of these points or will muddy the waters even further.

## Author Contributions

JM, TS, and RW are responsible for the concept of the article. JM together with TS wrote the manuscript and relied on fruitful discussions with RW and DM. DM supported by FH provided the update of the systematic map on genome editing of market-oriented traits and numerous references cited in the article. All authors have read and agreed to the published version of the manuscript.

## Conflict of Interest

The authors declare that the research was conducted in the absence of any commercial or financial relationships that could be construed as a potential conflict of interest.

## Supplementary Material

Supplementary Table 1 Tables with market-oriented genome editing applications as basis for the figures including 219 references to the literature, wherein the genome editing studies were identified.
