# Supplementary material for: Genome Edited Crops Touch the Market: A View on the Global Development and Regulatory Environment
Source: Front Plant Sci. 2020 Oct 9;11:586027. doi: 10.3389/fpls.2020.586027 (PMC7581933; doi:10.3389/fpls.2020.586027)
Supplement: Supplementary Table 1 — Tables with market-oriented genome editing applications as basis for the figures including 219 references to the literature, wherein the genome editing studies were identified. [file Data_Sheet_1.PDF]

Table 1: Genome editing in plants to modify agronomically relevant traits (1996 - June 2019).

Plant	Producer, Country	Trait	Spezifikation	Technique	Reference
Cotton	Anhui Agricultural University, China; Chinese Academy of Agricultural Sciences, China	Growth performance	Increased root growth under high- and low N-conditions	CRISPR/Cas9 SDN1	[3]
Cucumber	Chinese Academy of Agricultural Sciences, China	Growth performance	Only female flowers	CRISPR/Cas9 SDN1	[4]
Potato	Cellectis Plant Science, USA	Storage performance	Improved cold storage and frying conditions (reduced sugar/ reduced acrylamide)	TALENs SDN1	[5]
Kiwi	The New Zealand Institute for Plant & Food Research Limited, New Zealand	Growth performance	Compact growth, early flowering	CRISPR/Cas9 SDN1	[6]
Maize	Benson Hill Biosystems, USA	Yield increase	Improved photosynthesis efficiency	Meganuclease SDN3	[7]
Maize	University of Wisconsin, USA	Growth performance	Early flowering under long day conditions	CRISPR/Cas9 SDN1	[8]
Canola	Christian-Albrechts-Universität Kiel, Germany	Yield increase	Scatter resistance	CRISPR/Cas9 SDN1	[9]
Canola	Huazhong Agricultural University, China	Yield increase	Increased seed number/pod, higher grain weight	CRISPR/Cas9 SDN1	[10]
Canola	Huazhong Agricultural University,China	Growth performance	Changed leaf shape	CRISPR/Cas9 SDN1	[11]
Canola	Hunan Agricultural University, China; Universite de Strasbourg, France	Growth performance	Early flowering	CRISPR/Cas9 SDN1	[12]
Rice	Chinese Academy of Sciences, China	Yield increase	Increased seed number/panicle	CRISPR/Cas9 SDN1	[13, 14]
	National Rice Research Institute, China			CRISPR/Cas9 SDN1	[15]
	Wuhan Institute of Bioengineering, China			CRISPR/Cas9 SDN1	[16]
Rice	Chinese Academy of Sciences, China	Yield increase	Higher Grain size and weight / Thousand seed weight	CRISPR/Cas9 SDN1	[13]
	Anhui Academy of Agricultural Sciences, China			CRISPR/Cas9 SDN1	[17]
	Fudan University, China			CRISPR/Cas9 SDN1	[18]
	Yangzhou University, China			CRISPR/Cas9 SDN1	[19, 20]
	Agronomy College of Henan Agricultural University, China			CRISPR/Cas9 SDN1	[21]

Plant	Producer, Country	Trait	Spezifikation	Technique	Reference
	Chinese Academy of Agricultural Sciences, China; Yangzhou University, China			CRISPR/Cas9 SDN1	[22]
	Okayama University, Henan Agricultural University China			CRISPR/Cas9 SDN1	[23–25]
	University of Maryland, USA			CRISPR/Cas9 SDN1	[26]
Rice	Chinese Academy of Sciences, China	Growth performance	Larger plants, improved tillering, upright panicles, increased biomass	CRISPR/Cas9; SDN1	[13]
	Wuhan Institute of Bioengineering; Huazhong Agricultural University, China			CRISPR/Cas9 SDN1	[27]
	Sichuan Agricultural University, China			CRISPR/Cas9 SDN1	[28]
	Chinese Academy of Agricultural Sciences, China; Yangzhou University, China			CRISPR/Cas9 SDN1	[22]
Rice	Chinese Academy of Sciences, China<br>Rice Research Institute of Shenyang Agricultural University, Shenyang, China	Growth performance	Early flowering	CRISPR/Cas9 SDN1	[29, 30]
Rice	Chinese Academy of Agricultural Sciences, China; Jangsu Academy of Agricultural Sciences, China	Growth performance	Early ripening	CRISPR/Cas9 SDN1	[31]
Rice	King Abdullah University of Science and Technology, Saudi-Arabia	Growth performance	improved tillering, reduced plant height	CRISPR/Cas9 SDN1	[32]
Rice	Chinese Academy of Sciences, China; University of Chinese Academy of Sciences, China	Yield increase	Regulation of pollen growth	CRISPR/Cas9 SDN1	[33]
Rice	China Agricultural University, China	Storage performance	Longevity of seeds	TALENs SDN1	[34]
Rice	Nanjing Agricultural University, China	Yield increase	Grain yield, Regulation of seed-development	CRISPR/Cas9 SDN1	[35]
Rice	Anhui Academy of Agricultural Sciences, China	Yield increase	Longer penicles	CRISPR/Cas9 SDN1	[17]
Rice	Chinese Academy of Sciences, China	Growth performance	reduced plant height	BE SDN1	[36]
	Syngenta Biotechnology, China			CRISPR/Cas9 SDN1	[37]

Plant	Producer, Country	Trait	Spezifikation	Technique	Reference
Rice	Wuhan Institute of Bioengineering, China; Huazhong Agricultural University, China	Yield increase	Improved N-efficiency	CRISPR/Cas9 SDN1	[27]
Rice	Hunan Normal University, China	Growth performance	Dormancy regulation, stomata development, improved plant growth, abiotic Stress tolerance and senescence	CRISPR/Cas9 SDN1	[38]
Rice	Xiamen University, China	Growth performance	Red colored rice	CRISPR/Cas9 SDN1	[39]
Rice	Chinese Academy of Sciences, China	Growth performance	Increased leaf dispersion (increased surface)	CRISPR/Cas9 SDN1	[40]
Millet	Iowa State University, USA	Growth performance	bushy plants	CRISPR/Cas9 SDN1	[41]
Lettuce	University of California, USA	Yield increase	Increased germination under higher temp.	CRISPR/Cas9 SDN1	[42]
Soybean	Chinese Academy of Agricultural Sciences, China	Growth performance	late flowering	CRISPR/Cas9 SDN1	[43, 44]
Soybean	Chinese Academy of Agricultural Sciences, China	Growth performance/ Yield increase	late flowering under short-day conditions improved pod- and seed set/Plant	CRISPR/Cas9 SDN1	[45]
Soybean	University of Minnesota, USA	Growth performance	Changed petiole length	CRISPR/Cas9 SDN1	[46]
Tomato	National Food Research Institute, Japan<br>Bioscience, Wageningen Plant Research, The Netherlands	Yield increase	Fast ripping fruits	CRISPR/Cas9 SDN1<br>CRISPR/Cas9 SDN1	[47]<br>[48]
Tomato	University of Minnesota, USA	Growth performance	Bigger seedlings	TALENs SDN1	[49]
Tomato	Norwich Research Park, Great Britain	Growth performance	Dwarfism	CRISPR/Cas9 SDN1	[50]
Tomato	Cold Spring Harbor Laboratory, USA; Max Planck Institute for Plant Breeding Research, Germany; Université Paris-Scalay, France	Growth performance	Early flowering	CRISPR/Cas9 SDN1	[51]
Tomato	University of Florida, USA	Growth performance	Easier separation of the fruit from the stalk	CRISPR/Cas9 SDN1	[52]
Tomato	Cold Spring Harbor Laboratory, USA	Yield increase	Improved fruit size	CRISPR/Cas9 SDN1	[53]
Tomato	Cold Spring Harbor Laboratory, USA	Yield increase	Strongly branched inflorescences and formation of many flowers	CRISPR/Cas9 SDN1	[53]
Tomato	Weizmann Institute of Science, Israel	Growth performance	Yellow fruits	CRISPR/Cas9 SDN1	[54]

Plant	Producer, Country	Trait	Spezifikation	Technique	Reference
	Weizmann Institute of Science, Israel			CRISPR/Cas9 SDN3	[55]
	Agenzia Lucana per lo Sviluppo e l'Innovazione in Agricoltura, Italy			CRISPR/Cas9 SDN1	[56]
Tomato	Weizmann Institute of Science, Israel	Growth performance	Orange fruits	CRISPR/Cas9 SDN3	[55]
Tomato	Academy of Agriculture and Forestry Sciences; Chinese Academy of Sciences, China	Growth performance	Pink fruits	CRISPR/Cas9 SDN1	[57]
Wild strawberry	University of Maryland, USA;	Growth performance	Faster seedling development	CRISPR/Cas9 SDN1	[58]
Wheat	Kansas State University, USA; Norwich Research Park, GB	Yield increase	Larger grains, higher grain weight, higher thousand-grain weight	CRISPR/Cas9 SDN1	[59,]
	Kansas State University, USA			CRISPR/Cas9 SDN1	
	Chinese Academy of Sciences, China			CRISPR/Cas9 SDN1	[61]
Wheat	South Dakota State University, USA	Yield increase	Higher grain number per ear, increased grain weight per ear	CRISPR/Cas9 SDN1	[62]

BE: Base Editing
TALENs: Transcription Activator-Like Effector Nucleases
CRISPR/Cas9: Clustered Regularly Interspaced Short Palindromic Repeats/CRISPR associated protein 9
SDN: Site Directed Nucleases

Table 2: Genome editing to improve food or feed quality (1996 - June 2019).

Plant	Producer, Country	Trait	Specification	Technique	Reference
Field pennycress	Illinois State University, USA	Product quality	Changed oil composition	CRISPR/Cas9 SDN1	[63, 64]
Alfalfa	Calyxt, Inc., USA	Product quality	Reduced lignin content	TALENs SDN1	[65]
peanut	Guangdong Academy of Agricultural Sciences, China	Product quality	Increased oleic acid content, reduced linoleic acid content	TALENs SDN1	[66]
Millet	University of Nebraska, USA	Product quality	Improved digestibility of grain protein and modified lysine content	CRISPR/Cas9 SDN1	[67]
Potato	Calyxt, USA	Product quality	Non-browning	TALENs SDN1	[68]
Potato	Simplot Plant Science, USA	Product quality	Reduced black spotting	TALENs SDN1	[69]
Potato	RIKEN Center for Sustainable Resource Science, Japan; Chiba University, Japan	Product quality	Reduced glycoalcaloides	TALENs SDN1	[70]

Plant	Producer, Country	Trait	Specification	Technique	Reference
	Kobe University, Japan		Elimination of glycoalcaloides	CRISPR/Cas9 SDN1	[71]
Camelina	Montana State University, USA	Product quality	Higher oleic acid and α linolenic acid content	CRISPR/Cas9 SDN1	[72]
	University Nebraska, USA		Higher oleic acid content, lower fatty acid content	CRISPR/Cas9 SDN1	[73]
	Université Paris-Saclay, France		Higher oleic acid content, lower fatty acid content	CRISPR/Cas9 SDN1	[74]
	Kansas State University, USA		Reduced oil content	CRISPR/Cas9 SDN1	[75]
	Rothamsted Research, UK		Higher oleic acid content, lower fatty acid content	CRISPR/Cas9 SDN1	[76]
Maize	Du Pont Pioneer, USA;	Product quality	Waxycorn, modified starch composition	CRISPR/Cas9 SDN1	[77]
	Chinese Academy of Agricultural Sciences, China			CRISPR/Cas9 SDN1	[78]
Maize	Agrivida, USA	Product quality	Higher starch content in leaves and stems	Meganuc-lease SDN1	[79]
Maize	Chinese Academy of Agricultural Sciences, China	Product quality	Higher sugar content in grains	CRISPR/Cas9 SDN1	[80]
Cassava	ETH Zurich, Schweiz; University of Liege, Belgium	Product quality	modified starch composition	CRISPR/Cas9 SDN1	[81]
Agaricus	Penn State University, USA	Product quality	Non browning	CRISPR/Cas9 SDN1	[82]
Canola	Tamagawa University, Japan	Product quality	Changed oil composition	CRISPR/Cas9 SDN1	[83]
Rice	Chinese Academy of Sciences, China	Product quality	Scented rice	TALENs SDN1	[84]
	Chinese Academy of Agricultural Sciences, China; Yangzhou University, China			CRISPR/Cas9 SDN1	[22]
Rice	Chinese Academy of Agricultural Sciences, China; University of California, USA	Product quality	Changed starch content (increased amylose content)	CRISPR/Cas9 SDN1	[85]
Rice	Guangxi University, Jiangsu Academy of Agricultural Sciences, China	Product quality	Changed starch content (reduced amylose content)	CRISPR/Cas9 SDN1	[86, 87]
	University of Lleida-Agrotecnio Center, Spain				[88]
Rice	Huazhong Agricultural University, China	Product quality		CRISPR/Cas9 SDN1	[89]

Plant	Producer, Country	Trait	Specification	Technique	Reference
	Sun Yat-sen University, China		Reduction of ingredients harmful to health (arsenic content)	CRISPR/Cas9 SDN1	[90]
Rice	National Agriculture and Food Research Organization, Japan	Product quality	Changed oil composition	CRISPR/Cas9 SDN1	[91]
Rice	Université Montpellier, France	Product quality	Reduction of ingredients harmful to health (caesium content)	CRISPR/Cas9 SDN1	[92]
Rice	Hunan Agricultural University, Hunan Hybrid Rice Research Center, Normal University, China	Product quality	Reduction of ingredients harmful to health (cadmium content)	CRISPR/Cas9 SDN1	[93]
Rice	Chinese Academy of Sciences, Shanghai, China; Purdue University, West Lafayette, USA	Product quality	Waxy rice	CRISPR/Cas9 SDN1	[94]
Salvia	Second Military Medical University, China	Product quality	Reduction of phenolic acid content	CRISPR/Cas9 SDN1	[95]
Lettuce	Chinese Academy of Sciences, China	Product quality	Increased ascorbic acid content,<br>improved tolerance to oxidative stress	CRISPR/Cas9 SDN1	[96]
Lettuce	Ag Bio Division, USA	Product quality	Reduced/ slowed browning of leaves	CRISPR/Cas9 SDN1	[97]
Poppy	Cankiri Karatekin University, Türkei; Dokuz Eylul University, Turkey	Product quality	Reduced morphine and thebain content	CRISPR/Cas9 SDN1	[98]
Soybean	Cellectis plant science Inc., USA/Calyxt, USA<br><br>Jilin Agricultural University, China	Product quality	High oleic acid content,<br>low linoleic acid content	TALENs SDN1<br><br>CRISPR/Cas9 SDN1	[99–102]<br><br>[103]
Tomato	Agricultural Research Organization, Israel;<br><br>Tokushima University, Japan	Product quality	Seedless fruits	CRISPR/Cas9 SDN1<br><br>CRISPR/Cas9 SDN1	[104]<br><br>[105]
Tomato	University of Tsukuba, Japan;<br><br>China Agricultural University, China	Product quality	Increase of health-promoting ingredients (increased GABA content)	CRISPR/Cas9 SDN1<br><br>CRISPR/Cas9 SDN1	[106]<br><br>[107]
Tomato	China Agricultural University, China	Product quality	Increase of health-promoting ingredients (increased lycopene content)	CRISPR/Cas9 SDN1	[108]
Tomato	Xinjiang Academy of Agricultural Science, China	Product quality	Longer storage at room temperature	CRISPR/Cas9 SDN1	[109]
Wheat	Okayama University, Japan	Product quality	Longer seed dormancy (prevents germination before harvest)	CRISPR/Cas9 SDN1	[110]

Plant	Producer, Country	Trait	Specification	Technique	Reference
Wheat	Calyxt, Inc., USA	Product quality	Higher nutritional value	TALENs<br>SDN1	[111]
Wheat	Instituto de Agricultura Sostenible (IAS-CSIC), Spain; University of Minnesota, USA	Product quality	Reduced gluten content	CRISPR/Cas9<br>SDN1	[112]
Wheat (Durum)	Instituto de Agricultura Sostenible (IAS-CSIC), Spain; University of Minnesota, USA	Product quality	Reduced gluten content	CRISPR/Cas9<br>SDN1	[112]

TALENs: Transcription Activator-Like Effector Nucleases
CRISPR/Cas9: Clustered Regularly Interspaced Short Palindromic Repeats/CRISPR associated protein 9
SDN: Site Directed Nucleases

Table 3: Genome editing in plants to improve resistance/tolerance to biotic stress (1996 - June 2019).

Plant	Producer, Country	Trait	Specification	Technique	Reference
Banana	International Institute of Tropical Agriculture (IITA), Kenia	Virus resistance	Resistance against *Banana Streak Virus* (BSV)	CRISPR/Cas9<br>SDN1	[113]
Cotton	Chinese Academy of Sciences; Chinese Academy of agricultural Sciences, China	Fungi resistance	Resistance against *Verticillium dahlia*	CRISPR/Cas9<br>SDN1	[114]
Barley	Agricultural Biotechnology Institute, Hungary	Virus resistance	Resistance against *Wheat Dwarf Virus*	CRISPR/Cas9<br>SDN1	[115]
Grapefruit	University of Florida, USA;<br>Gannan Normal University, China	Resistance against bacteria	Citrus cancer resistance	CRISPR/Cas9<br>SDN1<br>CRISPR/Cpf1<br>SDN1	[116, 117]<br>[118]
Cucumber	Volcani Center, Israel	Virus resistance	Immunity to Cucumber Vein Yellowing Virus infection (Ipomovirus) and resistance to Potyvirus, Zucchini Yellow Mosaic Virus and Papaya Ring Spot Mosaic Virus-W.	CRISPR/Cas9<br>SDN1	[119]
cacao	Pennsylvania State University, USA	Fungi resistance	*Phytophthora tropicalis* resistance	CRISPR/Cas9<br>SDN1	[120]
Potato	Doka Gene Technologies Ltd;<br>Moscow State University, Russia<br>Hubei University, China; Max-Planck-Institut für Molekulare Plant physiology, Germany	Virus resistance	*Potato Virus Y* (PVY) resistance	CRISPR/Cas9<br>SDN1<br>CRISPR/Cas13a<br>SDN1	[121]<br>[122]
Maize	Du Pont Pioneer, USA	Fungi resistance	*Northern Leaf Blight* resistance	CRISPR/Cas9 (Cisgenesis)<br>SDN3	[123]
Cassava	Donald Danforth Plant Science Center, USA	Virus resistance	Resistance to brown streak disease	CRISPR/Cas9<br>SDN1	[124]

Plant	Producer, Country	Trait	Specification	Technique	Reference
Orange	Chinese Academy of Agricultural Sciences and National Center for Citrus Variety Improvement; Southwest University, China	Resistance against bacteria	Citrus cancer resistance	CRISPR/Cas9 SDN1	[125]
Canola	Yangzhou University, China	Fungi resistance	*Sclerotinia sclerotiorium* resistance	CRISPR/Cas9 SDN1	[126]
Rice	Chinese Academy of Agriculture, China	Fungi resistance	Resistance to rice browning	CRISPR/Cas9; SDN1	[127]
Rice	Huazhong Agricultural University, China	Fungi resistance	Resistance to rice browning, early earing	CRISPR/Cas9; SDN1	[128, 129]
Rice	Iowa State University, USA<br>IRD-CIRAD- Université, France<br>Iowa State University, USA<br>National University of Singapore, Singapure<br>Chinese Academy of Sciences, China<br>National Center for Plant Gene Research, China; Sichuan Agricultural University, China<br>Sichuan Agricultural University, China	Resistance against bacteria	Resistance against bacterial blight	CRISPR/Cas9; SDN1<br>TALENs SDN1<br>TALENs SDN1<br>TALENs SDN1<br>TALENs SDN1<br>CRISPR/Cas9; SDN1<br>CRISPR/Cas9 SDN1	[130]<br>[131]<br>[132] [133]<br>[134]<br>[129, 135]<br>[136]<br>[28]
Rice	Shanghai Jiao Tong University, China; Yunnan Academy of Agricultural Sciences, China	Resistance against bacteria	*Xanthomonas RS105* Resistance	TALENs SDN1	[137]
Rice	International Rice Research Institute (IRRI), Philippines	Virus resistance	*Rice-Tungro- Virus* resistance	CRISPR/Cas9 SDN1	[138]
Rice	South China Agricultural University, Guangzhou, China	Virus resistance	*Southern Rice Black-streaked Dwarf Virus* resistance	CRISPR/Cas13a SDN1	[139]
Tomato	Max Planck Institute for Developmental Biology, Germany; Norwich Research Park, UK	Fungi resistance	Powdery mildew resistance	CRISPR/Cas9 SDN1	[140]
Tomato	King Abdullah University of Science and Technology, Saudi-Arabia	Virus resistance	*Tomato Yellow Leaf Virus* resistance	CRISPR/Cas9 SDN1	[141]
Tomato	University of California, USA	Resistance against bacteria	Multiple resistance e.g. *P. syringae, P. capsici und Xanthomonas spp.*	CRISPR/Cas9 SDN1	[142]

Plant	Producer, Country	Trait	Specification	Technique	Reference
Tomato	Consejo Superior de Investigaciones Científicas, Spain	Resistance against bacteria	leaf and fruit blotch disease resistance	CRISPR/Cas9 SDN1	[143]
Grapevine	Northwest A&F University and Ministry of Agriculture, China	Fungi resistance	Gray mold resistance	CRISPR/Cas9 SDN1	[144]
Wheat	Chinese Academy of Sciences, China	Fungi resistance	Powdery mildew resistance	TALENs SDN1	[145]
	Chinese Academy of Sciences, China			CRISPR/Cas9 SDN1	[146]

TALENs: Transcription Activator-Like Effector Nucleases
CRISPR/Cas9: Clustered Regularly Interspaced Short Palindromic Repeats/CRISPR associated protein 9
SDN: Site Directed Nucleases

Table 4: Genome editing for the production of herbicide-tolerant plants (1996 – Juni 2019).

Plant	Producer, Country	Trait*	Specification	Technique	Reference
Cotton	Bayer CropScience N.V., Belgium	Herbicide tolerance	-	Meganuclease SDN3	[147]
Linum	Cibus, USA	Herbicide tolerance	-	CRISPR/Cas9 SDN1	[148]
Potato	Michigan State University, USA	Herbicide tolerance	-	CRISPR/Cas9, TALENs SDN2	[149]
Maize	DuPont Pioneer, USA	Herbicide tolerance	-	CRISPR/Cas9 SDN1, SDN2, SDN3	[150, 151]
	Dow AgroScience, USA			ZFN SDN3	[152]
	Pioneer Hi-Bred International, USA			ODM	[153, 154]
Cassava	Donald Danforth Plant Science Center, St. Louis, USA	Herbicide tolerance	-	CRISPR/Cas9 SDN3	[155]
Canola	Cibus, Canada; Cibus, USA;	Herbicide tolerance	-	ODM	[156]
	Bayer BioScience N.V., Belgium				[157]
Rice	Chinese Academy of Sciences, China	Herbicide tolerance	-	CRISPR/Cas9 SDN2	[158]
	Chinese Academy of Sciences, China; Huazhong Agricultural University, China; University of California San Diego, USA;			CRISPR/Cas9 SDN2	[159]
	Zhejiang University, China			TALENs SDN2	[160]
	Tohoku University, Japan			ODM	[161]

Plant	Producer, Country	Trait*	Specification	Technique	Reference
	Kobe University, Japan; University of Tsukuba, Japan; Chinese academy of Science, China			BE	[162–164]
	King Abdullah University of Science and Technology, Saudi-Arabia			CRISPR/Cas9 SDN2	[165]
Soybean	DuPont Pioneer, USA	Herbicide tolerance	-	CRISPR/Cas9 SDN2	[166]
				CRISPR/Cas9 SDN3	[167]
Tomato	GAFL Unit (Génétique et Amelioration des Fruits et Légumes), France	Herbicide tolerance	-	CRISPR/Cas9 SDN1	[168]
Watermelon	China Agricultural University, China; Beijing Academy of Agriculture and Forestry Sciences, China	Herbicide tolerance	-	CRISPR/Cas9 SDN1	[169]
Wheat	Chinese Academy of Sciences, China Agricultural University, China	Herbicide tolerance	-	BE	[170]

* no detailed classification of chemical substances
TALENs: Transcription Activator-Like Effector Nucleases
CRISPR/Cas9: Clustered Regularly Interspaced Short Palindromic Repeats/CRISPR associated protein 9
ZFN: Zinc-Finger Nuclease
ODM: Oligonucleotide-Directed Mutagenesis
SDN: Site Directed Nucleases
BE: Base Editing

Table 5: Genome editing in plants for industrial use (1996 - June 2019).

Plant	Producer, Country	Trait	Specification	Technique	Reference
Potato	Swedish University of Agricultural Sciences, Sweden	Product quality	Improved starch quality	CRISPR/Cas9 SDN1	[171]
	Université Rennes, France			CRISPR/Cas9 SDN1	[172]
	Tokyo University of Science, Japan			CRISPR/Cas9 SDN1	[173]
Dandelion	Fraunhofer Institute for Molecular Biology and Applied Ecology, Germany	Growth performance	Higher biomass of roots, taproots, increased rubber and inulin content	CRISPR/Cas9	[174]
Millet	Noble Research Institute, USA	Product quality	Reduction of lignin	CRISPR/Cas9 SDN1	[175]
Tobacco	TU Dortmund University, Germany	Product quality	Reduced nicotine content	CRISPR/Cas9 SDN1	[176]

Plant	Producer, Country	Trait	Specification	Technique	Reference
Sugar cane	University of Florida, USA	Product quality	Reduction of lignin	TALENs<br>SDN1	[177, 178]

CRISPR/Cas9: Clustered Regularly Interspaced Short Palindromic Repeats/CRISPR associated protein 9
TALENs: Transcription Activator-Like Effector Nucleases
SDN: Site Directed Nucleases

Table 6: Genome editing in plants to improve tolerance to abiotic stress (1996 - June 2019).

Plant	Producer, Country	Trait	Specification	Technique	Reference
Potato	Doka Gene Technologies Ltd;<br>Moscow State University, Russia	Salt tolerance	-	CRISPR/Cas9<br>SDN1	[121]
Maize	Ghent University, Belgium; Center for Plant Systems Biology, Belgium; Jomo Kenyatta University of Agriculture and Technology, Kenia	Drough tolerance	-	CRISPR/Cas9<br>SDN1	[179]
	DuPont Pioneer, USA			CRISPR/Cas9<br>SDN3	[167, 180]
Rice	Anhui Academy of Agricultural Sciences, China	Salt tolerance	-	CRISPR/Cas9<br>SDN1	[181]
	Huazhong Agricultural University, China			CRISPR/Cas9<br>SDN1	[182]
Soybean	USDA-ARS, USA	Drough- and Salt tolerance	-	CRISPR/Cas9<br>SDN1	[183]
Wheat	Montana State University, USA	Drough tolerance	-	CRISPR/Cas9<br>SDN1	[184]

CRISPR/Cas9: Clustered Regularly Interspaced Short Palindromic Repeats/CRISPR associated protein 9
SDN: Site Directed Nucleases

Table 7: Applied genome editing on different traits, thus assignment to several categories (1996 - June 2019).

Plant	Producer, Country	Trait	Specification	Technique	Reference
Cabbage	Ministry of Education; Southwest University, China	Growth performance	Self-incompatibility, male sterility	CRISPR/Cas9<br>SDN1	[185]
Maize	Dow AgroScience, USA	Product quality, Herbicide tolerance	reduced Phytat-production, Herbicide tolerance	ZFN<br>SDN3	[186, 187]
Physalis	Cold Spring Harbor; The Boyce Thompson Institute, USA;	Growth performance<br>Yield increase	compact growth, increased flower production, bigger fruits	CRISPR/Cas9<br>SDN1	[188]
Rice	Chinese Academy of Sciences, China	Growth performance	Heterozygosity, clonal Seed propagation	CRISPR/Cas9	[189]

Plant	Producer, Country	Trait	Specification	Technique	Reference
Wild Tomato	Chinese Academy of Sciences, China	Growth performance, Yield increase, Product quality	Day lenght sensibility, shoot architecture, flower- and fruit production, higher Vitamin C-content	CRISPR/Cas9 SDN1	[190]
Wild Tomato	Universität Münster, Germany; Universidade de Sao Paulo, Brasil	Growth performance, Yield increase, Product quality	changed fruit shape, compact growth, increased fruit number, larger fruits, increased constitutional ingredients (higher Lycopincontent)	CRISPR/Cas9 SDN1	[191]

CRISPR/Cas9: Clustered Regularly Interspaced Short Palindromic Repeats/CRISPR associated protein 9
SDN: Site Directed Nucleases
ZFN: Zink-Finger Nukleasen

Table 8: Genome editing to improve plant breeding (1996 - June 2019).

Plant	Producer, Country	Trait	Specification	Technique	Reference
Potato	Chinese Academy of Agricultural Sciences, China	Growth performance	Self-incompatibility	CRISPR/Cas9 SDN1	[192]
	Michigan State University, USA			CRISPR/Cas9 SDN1	[193]
Maize	University of Science and Technology Beijing, China; Beijing Solidwill Sci-Tech Co. Ltd, China	Growth performance	male sterility	CRISPR/Cas9 SDN1	[194]
	Chinese Academy of Sciences, China			CRISPR/Cas9 SDN1	[23, 195, 196]
Maize	Syngenta Seeds, USA	Growth performance	Haploid induction	TALENs SDN1	[197]
	Syngenta Seeds, USA			CRISPR/Cas9 SDN1	[198]
	Chinese Academy of Agricultural Sciences, South China Agricultural University (Guangzhou),China Agricultural University (Beijing), China			CRISPR/Cas9 SDN1	[199, 200]
Rice	Kyung Hee University, South Korea	Growth performance	Male sterility	CRISPR/Cas9 SDN1	[201]
	Shanghai Jiao Tong University, China			CRISPR/Cas9 SDN1	[202, 203]
	South China Agricultural University, China			CRISPR/Cas9 SDN1	[204, 205]
	Sichuan Agricultural University, China			CRISPR/Cas9 SDN1	[206, 207]
	National Rice Research Institute, China			CRISPR/Cas9 SDN1	[208]
	Chinese Academy of Agricultural Sciences, China			CRISPR/Cas9 SDN1	[209]

Plant	Producer, Country	Trait	Specification	Technique	Reference
Rice	University of California; Iowa State University, USA	Growth performance	Asexual reproduction	CRISPR/Cas9 SDN1	[210]
Wheat	DuPont Pioneer, USA<br>The University of Adelaide, Australia	Growth performance	Male sterility	CRISPR/Cas9 SDN1<br>CRISPR/Cas9 SDN1	[211]<br>[212]

CRISPR/Cas9: Clustered Regularly Interspaced Short Palindromic Repeats/CRISPR associated protein 9
SDN: Site Directed Nucleases

Table 9: Genome editing in ornamental plants (1996 - June 2019).

Plant	Producer, Country	Trait	Specification	Technique	Reference
Japanese morning glory	University of Tsukuba, Japan	Growth performance	Changed flower color	CRISPR/Cas9 SDN1	[213, 214]
Japanese morning glory	University of Tsukuba, Japan	Growth performance	increased flowering time	CRISPR/Cas9 SDN1	[215]
Orchid	Chinese Academy of Sciences, China	Product quality	Reduction of Lignocellulose (lignification)	CRISPR/Cas9 SDN1	[216]
Petunia	Kyungpook National University, South Korea	Growth performance	increased flowering time of individual flowers	CRISPR/Cas9 SDN1	[217]
Tobaccok	China Tobacco Gene Research Center, China	Growth performance	Auxin biosynthesis	CRISPR/Cas9 SDN1	[218]
Torenia	Iwate Biotechnology Research Center, Japan	Growth performance	Changed flower color	CRISPR/Cas9 SDN1	[219]

CRISPR/Cas9: Clustered Regularly Interspaced Short Palindromic Repeats/CRISPR associated protein 9
SDN: Site Directed Nuclease

References

1. Modrzejewski D, Hartung F, Sprink T, Krause D, Kohl C, Wilhelm R. What is the available evidence for the range of applications of genome-editing as a new tool for plant trait modification and the potential occurrence of associated off-target effects: a systematic map. Environ Evid. 2019;8:11. doi:10.1186/s13750-019-0171-5.
2. Modrzejewski D, Hartung F, Sprink T, Krause D, Kohl C, Schiemann J, Wilhelm R. What is the available evidence for the application of genome editing as a new tool for plant trait modification and the potential occurrence of associated off-target effects: a systematic map protocol. Environ Evid. 2018;7:11. doi:10.1186/s13750-018-0130-6.
3. Wang Y, Meng Z, Liang C, Meng Z, Wang Y, Sun G, et al. Increased lateral root formation by CRISPR/Cas9-mediated editing of arginase genes in cotton. Sci China Life Sci. 2017;60:524–7. doi:10.1007/s11427-017-9031-y.
4. Hu B, Li D, Liu X, Qi J, Gao D, Zhao S, et al. Engineering Non-transgenic Gynoecious Cucumber Using an Improved Transformation Protocol and Optimized CRISPR/Cas9 System. Mol Plant. 2017;10:1575–8. doi:10.1016/j.molp.2017.09.005.
5. Clasen BM, Stoddard TJ, Luo S, Demorest ZL, Li J, Cedrone F, et al. Improving cold storage and processing traits in potato through targeted gene knockout. Plant Biotechnol J. 2016;14:169–76. doi:10.1111/pbi.12370.
6. Varkonyi-Gasic E, Wang TC, Voogd C, Jeon S, Drummond RSM, Gleave AP, Allan AC.

Mutagenesis of kiwifruit CENTRORADIALIS-like genes transforms a climbing woody perennial with long juvenility and axillary flowering into a compact plant with rapid terminal flowering. 1467-7644. 2019;17:869–80. doi:10.1111/pbi.13021.
7. United States Department of Agriculture (USDA). 2015. https://www.aphis.usda.gov/biotechnology/downloads/reg_loi/15-013-01air.pdf. Accessed 25 Aug 2018.
8. Huang C, Sun HY, Xu DY, Chen QY, Liang YM, Wang XF, et al. ZmCCT9 enhances maize adaptation to higher latitudes. 0027-8424. 2018;115:E334-E341. doi:10.1073/pnas.1718058115.
9. Braatz J, Harloff H-J, Mascher M, Stein N, Himmelbach A, Jung C. CRISPR-Cas9 Targeted Mutagenesis Leads to Simultaneous Modification of Different Homoeologous Gene Copies in Polyploid Oilseed Rape (Brassica napus). Plant Physiol. 2017;174:9. doi:10.1104/pp.17.00426.
10. Yang Y, Zhu KY, Li HL, Han SQ, Meng QW, Khan SU, et al. Precise editing of CLAVATA genes in Brassica napus L. regulates multilocular silique development. 1467-7644. 2018;16:1322–35. doi:10.1111/pbi.12872.
11. Hu LM, Zhang H, Yang QY, Meng QW, Han SQ, Nwafor CC, et al. Promoter variations in a homeobox gene, BnA10.LMI1, determine lobed leaves in rapeseed (Brassica napus L.). Theor Appl Genet. 2018;131:2699–708. doi:10.1007/s00122-018-3184-5.
12. Jiang L, Li DH, Jin L, Ruan Y, Shen WH, Liu CL. Histone lysine methyltransferases BnaSDG8.A and BnaSDG8.C are involved in the floral transition in Brassica napus. Plant J. 2018;95:672–85. doi:10.1111/tpj.13978.
13. Li M, Li X, Zhou Z, Wu P, Fang M, Pan X, et al. Reassessment of the Four Yield-related Genes Gn1a, DEP1, GS3, and IPA1 in Rice Using a CRISPR/Cas9 System. Front Plant Sci. 2016;7:1–13. doi:10.3389/fpls.2016.00377.
14. Huang LY, Zhang R, Huang GF, Li YX, Melaku G, Zhang SL, et al. Developing superior alleles of yield genes in rice by artificial mutagenesis using the CRISPR/Cas9 system. Crop J. 2018;6:475–81. doi:10.1016/j.cj.2018.05.005.
15. Shen L, Wang C, Fu Y, Wang J, Liu Q, Zhang X, et al. QTL editing confers opposing yield performance in different rice varieties. J Integr Plant Biol 2016. doi:10.1111/jipb.12501.

16. Wang J, Wu BW, Lu K, Wei Q, Qian JJ, Chen YP, Fang ZM. The Amino Acid Permease 5 (OsAAP5) Regulates Tiller Number and Grain Yield in Rice. 0032-0889. 2019;180:1031–45. doi:10.1104/pp.19.00034.
17. Xu R, Yang Y, Qin R, Li H, Qiu C, Li L, et al. Rapid improvement of grain weight via highly efficient CRISPR/Cas9-mediated multiplex genome editing in rice. J Genet Genomics. 2016;43:529–32. doi:10.1016/j.jgg.2016.07.003.
18. Hu Z, Lu S-J, Wang M-J, He H, Le Sun, Wang H, et al. A Novel QTL q TGW3 Encodes the GSK3/SHAGGY-Like Kinase OsGSK5/OsSK41 that Interacts with OsARF4 to Negatively Regulate Grain Size and Weight in Rice. Mol Plant. 2018;11:736–49. doi:10.1016/j.molp.2018.03.005.
19. Shen Lan, Li Jian, Fu Yaping, Wang Junjie, Hua Yufeng, Jiao Xiaozhen, Yan Changjie, Wang Kejian. Orientation Improvement of Grain Length and Grain Number in Rice by Using CRISPR/Cas9 System 2017. doi:10.16819/j.1001-7216.2017.7029.
20. Miao J, Yang ZF, Zhang DP, Wang YZ, Xu MB, Zhou LH, et al. Mutation of RGG2, which encodes a type B heterotrimeric G protein gamma subunit, increases grain size and yield production in rice. 1467-7644. 2019;17:650–64. doi:10.1111/pbi.13005.
21. Ji X, Li F, Yan Y, Sun HZ, Zhang J, Li JZ, et al. CRISPR/Cas9 System-Based Editing of Phytochrome-Interacting Factor OsPIL15 2017. doi:10.3864/j.issn.0578-1752.2017.15.002.

22. Shen L, Hua Y, Fu Y, Li J, Liu Q, Jiao X, et al. Rapid generation of genetic diversity by multiplex CRISPR/Cas9 genome editing in rice. Sci China Life Sci. 2017;60:506–15. doi:10.1007/s11427-017-9008-8.
23. Chiou WY, Kawamoto T, Himi E, Rikiishi K, Sugimoto M, Hayashi-Tsugane M, et al. LARGE GRAIN Encodes a Putative RNA-Binding Protein that Regulates Spikelet Hull Length in Rice. Plant Cell Physiol. 2019;60:503–15. doi:10.1093/pcp/pcz014.
24. Ji X, Du Y, Li F, Sun H, Zhang J, Li J, et al. The basic helix-loop-helix transcription factor, OsPIL15, regulates grain size via directly targeting a purine permease gene OsPUP7 in rice 2019. doi:10.1111/pbi.13075.
25. Ma XS, Feng FJ, Zhang Y, Elesawi IE, Xu K, Li TF, et al. A novel rice grain size gene OsSNB was identified by genome-wide association study in natural population. Plos Genet. 2019;15.
26. Zhou JP, Xin XH, He Y, Chen HQ, Li Q, Tang X, et al. Multiplex QTL editing of grainrelated
genes improves yield in elite rice varieties. Plant Cell Rep. 2019;38:475–85. doi:10.1007/s00299-018-2340-3.
27. Lu K, Wu B, Wang J, Zhu W, Nie H, Qian J, et al. Blocking amino acid transporter OsAAP3 improves grain yield by promoting outgrowth buds and increasing tiller number in rice. Plant Biotechnol J. 2018;50:1416. doi:10.1111/pbi.12907.
28. Liao Y, Bai Q, Xu P, Wu T, Guo D, Peng Y, et al. Mutation in Rice Abscisic Acid2 Results in Cell Death, Enhanced Disease-Resistance, Altered Seed Dormancy and Development. Front. Plant Sci. 2018;9:1248. doi:10.3389/fpls.2018.00405.
29. Zeng LP, Liu X, Zhou ZZ, Li DY, Zhao XF, Zhu LH, et al. Identification of a G2-like transcription factor, OsPHL3, functions as a negative regulator of flowering in rice by coexpression
and reverse genetic analysis. Bmc Plant Biol. 2018;18.
30. Cui Y, Zhu MM, Xu ZJ, Xu Q. Assessment of the effect of ten heading time genes on reproductive transition and yield components in rice using a CRISPR/Cas9 system. Theor Appl Genet. 2019;132:1887–96. doi:10.1007/s00122-019-03324-1.
31. Li X, Zhou W, Ren Y, Tian X, Lv T, Wang Z, et al. High-efficiency breeding of earlymaturing
rice cultivars via CRISPR/Cas9-mediated genome editing. J Genet Genomics. 2017;44:175–8. doi:10.1016/j.jgg.2017.02.001.
32. Butt H, Jamil M, Wang JY, Al-Babili S, Mahfouz M. Engineering plant architecture via CRISPR/Cas9-mediated alteration of strigolactone biosynthesis. Bmc Plant Biol. 2018;18.
27
33. Liu L, Zheng C, Kuang B, Wei L, Yan L, Wang T. Receptor-Like Kinase RUPO Interacts with Potassium Transporters to Regulate Pollen Tube Growth and Integrity in Rice. PLoS Genet. 2016;12:e1006085. doi:10.1371/journal.pgen.1006085.
34. Ma L, Zhu F, Li Z, Zhang J, Li X, Dong J, Wang T. TALEN-Based Mutagenesis of Lipoxygenase LOX3 Enhances the Storage Tolerance of Rice (Oryza sativa) Seeds. PLoS ONE. 2015;10:e0143877. doi:10.1371/journal.pone.0143877.
35. Yuan J, Chen S, Jiao W, Wang L, Wang L, Ye W, et al. Both maternally and paternally imprinted genes regulate seed development in rice. New Phytol. 2017;216:373–87. doi:10.1111/nph.14510.
36. Lu Y, Zhu J-K. Precise Editing of a Target Base in the Rice Genome Using a Modified CRISPR/Cas9 System. Mol Plant. 2017;10:523–5. doi:10.1016/j.molp.2016.11.013.
37. Wang Y, Geng L, Yuan M, Wei J, Jin C, Li M, et al. Deletion of a target gene in Indica rice via CRISPR/Cas9. Plant Cell Rep. 2017;36:1333–43. doi:10.1007/s00299-017-2158-4.
38. Huang Y, Guo Y, Liu Y, Zhang F, Wang Z, Wang H, et al. 9-cis-Epoxycarotenoid

Dioxygenase 3 Regulates Plant Growth and Enhances Multi-Abiotic Stress Tolerance in Rice. Front. Plant Sci. 2018;9:1248. doi:10.3389/fpls.2018.00162.
39. Zhu Y, Lin Y, Chen S, Liu H, Chen Z, Fan M, et al. CRISPR/Cas9-mediated functional recovery of the recessive rc allele to develop red rice. 1467-7644 2019. doi:10.1111/pbi.13125.
40. Qu L, Lin LB, Xue HW. Rice miR394 suppresses leaf inclination through targeting an Fbox
gene, LEAF INCLINATION 4. J Integr Plant Biol. 2019;61:406–16. doi:10.1111/jipb.12713.
41. Liu Y, Merrick P, Zhang Z, Ji C, Yang B, Fei S-z. Targeted mutagenesis in tetraploid switchgrass (Panicum virgatum L.) using CRISPR/Cas9. Plant Biotechnol J. 2018;16:381–93. doi:10.1111/pbi.12778.
42. Bertier LD, Ron M, Huo H, Bradford KJ, Britt AB, Michelmore RW. High-Resolution Analysis of the Efficiency, Heritability, and Editing Outcomes of CRISPR/Cas9-Induced Modifications of NCED4 in Lettuce (Lactuca sativa). G3 (Bethesda). 2018;8:1513–21. doi:10.1534/g3.117.300396.
43. Cai YP, Chen L, Liu XJ, Guo C, Sun S, Wu CX, et al. CRISPR/Cas9-mediated targeted mutagenesis of GmFT2a delays flowering time in soya bean. 1467-7644. 2018;16:176–85. doi:10.1111/pbi.12758.
44. Cai YP, Chen L, Sun S, Wu CX, Yao WW, Jiang BJ, et al. CRISPR/Cas9-Mediated Deletion of Large Genomic Fragments in Soybean. Int J Mol Sci. 2018;19.
45. Cai Y, Wang L, Chen L, Wu T, Liu L, Sun S, et al. Mutagenesis of GmFT2a and GmFT5a mediated by CRISPR/Cas9 contribute for expanding the regional adaptability of soybean. 1467-7644 2019. doi:10.1111/pbi.13199.
46. Petiol length. APHIS-Am I Regulated. https://www.aphis.usda.gov/biotechnology/downloads/reg_loi/19-077-01_air_inquiry.pdf.
47. Ito Y, Nishizawa-Yokoi A, Endo M, Mikami M, Toki S. CRISPR/Cas9-mediated mutagenesis of the RIN locus that regulates tomato fruit ripening. Biochem Biophys Res Commun. 2015;467:76–82. doi:10.1016/j.bbrc.2015.09.117.
48. Wang RF, Tavano ECD, Lammers M, Martinelli AP, Angenent GC, Maagd RA de. Reevaluation
of transcription factor function in tomato fruit development and ripening with CRISPR/Cas9-mutagenesis. Sci Rep-Uk. 2019;9.
49. Lor VS, Starker CG, Voytas DF, Weiss D, Olszewski NE. Targeted mutagenesis of the tomato PROCERA gene using transcription activator-like effector nucleases. Plant Physiol. 2014;166:1288–91. doi:10.1104/pp.114.247593.

50. Tomlinson L, Yang Y, Emenecker R, Smoker M, Taylor J, Perkins S, et al. Using CRISPR/Cas9 genome editing in tomato to create a gibberellin-responsive dominant dwarf DELLA allele. 1467-7644. 2019;17:132–40. doi:10.1111/pbi.12952.
51. Soyk S, Müller NA, Park SJ, Schmalenbach I, Jiang K, Hayama R, et al. Variation in the flowering gene SELF PRUNING 5G promotes day-neutrality and early yield in tomato. Nat Genet. 2017;49:162–8. doi:10.1038/ng.3733.
52. United States Department of Agriculture (USDA). 2018. https://www.aphis.usda.gov/biotechnology/downloads/reg_loi/18-051-01_air_response_signed.pdf. Accessed 25 Aug 2018.
53. Rodríguez-Leal D, Lemmon ZH, Man J, Bartlett ME, Lippman ZB. Engineering Quantitative Trait Variation for Crop Improvement by Genome Editing. Cell. 2017;171:470-480.e8. doi:10.1016/j.cell.2017.08.030.
54. Filler Hayut S, Melamed Bessudo C, Levy AA. Targeted recombination between homologous chromosomes for precise breeding in tomato. Nat Commun. 2017;8:15605.

doi:10.1038/ncomms15605.
55. Dahan-Meir T, Filler-Hayut S, Melamed-Bessudo C, Bocobza S, Czosnek H, Aharoni A, Levy AA. Efficient in planta gene targeting in tomato using geminiviral replicons and the CRISPR/Cas9 system. The Plant Journal. 2018;95:5–16. doi:10.1111/tpj.13932.
56. D'Ambrosio C, Stigliani AL, Giorio G. CRISPR/Cas9 editing of carotenoid genes in tomato. Transgenic Res. 2018;27:367–78. doi:10.1007/s11248-018-0079-9.
57. Deng L, Wang H, Sun C, Li Q, Jiang H, Du M, et al. Efficient generation of pink-fruited tomatoes using CRISPR/Cas9 system. J Genet Genomics. 2018;45:51–4. doi:10.1016/j.jgg.2017.10.002.
58. Zhou J, Wang G, Liu Z. Efficient genome editing of wild strawberry genes, vector development and validation. Plant Biotechnol J. 2018;166:1292. doi:10.1111/pbi.12922.
59. Wang W, Pan Q, He F, Akhunova A, Chao S, Trick H, Akhunov E. Transgenerational CRISPR-Cas9 Activity Facilitates Multiplex Gene Editing in Allopolyploid Wheat. The CRISPR Journal. 2018;1:65–74. doi:10.1089/crispr.2017.0010.
60. Wang W, Simmonds J, Pan Q, Davidson D, He F, Battal A, et al. Gene editing and mutagenesis reveal inter-cultivar differences and additivity in the contribution of TaGW2 homoeologues to grain size and weight in wheat 2018. doi:10.1007/s00122-018-3166-7.
61. Zhang Y, Li D, Zhang D, Zhao X, Cao X, Dong L, et al. Analysis of the functions of TaGW2 homoeologs in wheat grain weight and protein content traits. The Plant Journal. 2018;94:857–66. doi:10.1111/tpj.13903.
62. Zhang Z, Hua L, Gupta A, Tricoli D, Edwards KJ, Yang B, Li W. Development of an Agrobacterium-delivered CRISPR/Cas9 system for wheat genome editing 2019. doi:10.1111/pbi.13088.
63. United States Department of Agriculture (USDA). 2018. https://www.aphis.usda.gov/biotechnology/downloads/reg_loi/18-036-01_a1_air_inquiry_cbidel.pdf. Accessed 25 Aug 2018.
64. McGinn M, Phippen WB, Chopra R, Bansal S, Jarvis BA, Phippen ME, et al. Molecular tools enabling pennycress (Thlaspi arvense) as a model plant and oilseed cash cover crop. 1467-7644. 2019;17:776–88. doi:10.1111/pbi.13014.
65. United States Department of Agriculture (USDA). 2017. https://www.aphis.usda.gov/biotechnology/downloads/reg_loi/17-038-02_air_inquiry_cbidel.pdf. Accessed 25 Aug 2018.
66. Wen SJ, Liu H, Li XY, Chen XP, Hong YB, Li HF, et al. TALEN-mediated targeted mutagenesis of fatty acid desaturase 2 (FAD2) in peanut (Arachis hypogaea L.) promotes the accumulation of oleic acid. Plant Mol Biol. 2018;97:177–85. doi:10.1007/s11103-018-0731-z.

67. Li AX, Jia SG, Yobi A, Ge ZX, Sato SJ, Zhang C, et al. Editing of an Alpha-Kafirin Gene Family Increases Digestibility and Protein Quality in Sorghum. 0032-0889. 2018;177:1425–38. doi:10.1104/pp.18.00200.
68. United States Department of Agriculture (USDA). 2016. https://www.aphis.usda.gov/biotechnology/downloads/reg_loi/16-090-01_air_inquiry_cbidel.pdf. Accessed 25 Aug 2018.
69. United States Department of Agriculture (USDA). 2016. https://www.aphis.usda.gov/biotechnology/downloads/reg_loi/16-320-01_air_inquiry.pdf. Accessed 25 Aug 2018.
70. Sawai S, Ohyama K, Yasumoto S, Seki H, Sakuma T, Yamamoto T, et al. Sterol side chain reductase 2 is a key enzyme in the biosynthesis of cholesterol, the common precursor of toxic steroidal glycoalkaloids in potato. Plant Cell. 2014;26:3763–74. doi:10.1105/tpc.114.130096.
71. Nakayasu M, Akiyama R, Lee HJ, Osakabe K, Osakabe Y, Watanabe B, et al. Generation

of α-solanine-free hairy roots of potato by CRISPR/Cas9 mediated genome editing of the St16DOX gene. Plant Physiol Biochem 2018. doi:10.1016/j.plaphy.2018.04.026.
72. Ozseyhan ME, Kang J, Mu X, Lu C. Mutagenesis of the FAE1 genes significantly changes fatty acid composition in seeds of Camelina sativa. Plant Physiol Biochem. 2018;123:1–7. doi:10.1016/j.plaphy.2017.11.021.
73. Jiang WZ, Henry IM, Lynagh PG, Comai L, Cahoon EB, Weeks DP. Significant enhancement of fatty acid composition in seeds of the allohexaploid, Camelina sativa, using CRISPR/Cas9 gene editing. Plant Biotechnol J. 2017;15:648–57. doi:10.1111/pbi.12663.
74. Morineau C, Bellec Y, Tellier F, Gissot L, Kelemen Z, Nogué F, Faure J-D. Selective gene dosage by CRISPR-Cas9 genome editing in hexaploid Camelina sativa. Plant Biotechnol J. 2017;15:729–39. doi:10.1111/pbi.12671.
75. Aznar-Moreno JA, Durrett TP. Simultaneous Targeting of Multiple Gene Homeologs to Alter Seed Oil Production in Camelina sativa. Plant Cell Physiol. 2017;58:1260–7. doi:10.1093/pcp/pcx058.
76. Rothamsted Research. 2019. https://gmoinfo.jrc.ec.europa.eu/gmp_report.aspx?CurNot=B/GB/19/R08/01. Accessed 28 Oct 2019.
77. United States Department of Agriculture (USDA). 2015. https://www.aphis.usda.gov/biotechnology/downloads/reg_loi/15-352-01_air_inquiry_cbidel.pdf. Accessed 25 Aug 2018.
78. Qi X, Le Dong, Liu C, Mao L, Liu F, Zhang X, et al. Systematic identification of endogenous RNA polymerase III promoters for efficient RNA guide-based genome editing technologies in maize. The Crop Journal. 2018;6:314–20. doi:10.1016/j.cj.2018.02.005.
79. United States Department of Agriculture (USDA). 2015. https://www.aphis.usda.gov/biotechnology/downloads/reg_loi/15-078-02_air_inquiry.pdf. Accessed 25 Aug 2018.
80. Dong L, Qi X, Zhu J, Liu C, Zhang X, Cheng B, et al. Supersweet and waxy: meeting the diverse demands for specialty maize by genome editing. 1467-7644 2019. doi:10.1111/pbi.13144.
81. Bull SE, Seung D, Chanez C, Mehta D, Kuon JE, Truernit E, et al. Accelerated ex situ breeding of GBSS- and PTST1-edited cassava for modified starch. Sci Adv. 2018;4.
82. United States Department of Agriculture (USDA). 2015. https://www.aphis.usda.gov/biotechnology/downloads/reg_loi/15-321-01_air_inquiry.pdf. Accessed 25 Aug 2018.

83. Okuzaki A, Ogawa T, Koizuka C, Kaneko K, Inaba M, Imamura J, Koizuka N. CRISPR/Cas9-mediated genome editing of the fatty acid desaturase 2 gene in Brassica napus. Plant Physiol Biochem 2018. doi:10.1016/j.plaphy.2018.04.025.
84. Shan Q, Zhang Y, Chen K, Zhang K, Gao C. Creation of fragrant rice by targeted knockout of the OsBADH2 gene using TALEN technology. Plant Biotechnol J. 2015;13:791–800. doi:10.1111/pbi.12312.
85. Sun Y, Jiao G, Liu Z, Zhang X, Li J, Guo X, et al. Generation of High-Amylose Rice through CRISPR/Cas9-Mediated Targeted Mutagenesis of Starch Branching Enzymes. Front Plant Sci. 2017;8:1–15. doi:10.3389/fpls.2017.00298.
86. Han Y, Luo DJ, Usman B, Nawaz G, Zhao N, Liu F, Li RB. Development of High Yielding Glutinous Cytoplasmic Male Sterile Rice (Oryza sativa L.) Lines through CRISPR/Cas9 Based Mutagenesis of Wx and TGW6 and Proteomic Analysis of Anther. Agronomy-Basel. 2018;8.
87. Fei YY, Yang J, Wang FQ, Fan FJ, Li WQ, Wang J, et al. Production of Two Elite

Glutinous Rice Varieties by Editing Wx Gene. Rice Sci. 2019;26:118–24. doi:10.1016/j.rsci.2018.04.007.
88. Pérez L, Soto E, Farré G, Juanos J, Villorbina G, Bassie L, et al. CRISPR/Cas9 mutations in the rice Waxy/GBSSI gene induce allele-specific and zygosity-dependent feedback effects on endosperm starch biosynthesis 2019. doi:10.1007/s00299-019-02388-z.
89. Ye Y, Li P, Xu T, Zeng L, Cheng D, Yang M, et al. OsPT4 Contributes to Arsenate Uptake and Transport in Rice. Front. Plant Sci. 2017;8:311. doi:10.3389/fpls.2017.02197.
90. Wang F-Z, Chen M-X, Yu L-J, Xie L-J, Yuan L-B, Qi H, et al. OsARM1, an R2R3 MYB Transcription Factor, Is Involved in Regulation of the Response to Arsenic Stress in Rice. Front Plant Sci. 2017;8:1868. doi:10.3389/fpls.2017.01868.
91. Abe K, Araki E, Suzuki Y, Toki S, SAIKA H. Production of high oleic/low linoleic rice by genome editing. Plant Physiol Biochem 2018. doi:10.1016/j.plaphy.2018.04.033.
92. Nieves-Cordones M, Mohamed S, Tanoi K, Kobayashi NI, Takagi K, Vernet A, et al. Production of low-Cs+ rice plants by inactivation of the K+ transporter OsHAK1 with the CRISPR-Cas system. Plant J. 2017;92:43–56. doi:10.1111/tpj.13632.
93. Tang L, Mao B, Li Y, Lv Q, Zhang L, Chen C, et al. Knockout of OsNramp5 using the CRISPR/Cas9 system produces low Cd-accumulating indica rice without compromising yield. Sci Rep. 2017;7:14438. doi:10.1038/s41598-017-14832-9.
94. Zhang J, Zhang H, Botella JR, Zhu J-K. Generation of new glutinous rice by CRISPR/Cas9-targeted mutagenesis of the Waxy gene in elite rice varieties. J Integr Plant Biol. 2018;60:369–75. doi:10.1111/jipb.12620.
95. Zhou Z, Tan H, Li Q, Chen J, Gao S, Wang Y, et al. CRISPR/Cas9-mediated efficient targeted mutagenesis of RAS in Salvia miltiorrhiza. Phytochemistry. 2018;148:63–70. doi:10.1016/j.phytochem.2018.01.015.
96. Zhang HW, Si XM, Ji X, Fan R, Liu JX, Chen KL, et al. Genome editing of upstream open reading frames enables translational control in plants. Nat Biotechnol. 2018;36:894-+. doi:10.1038/nbt.4202.
97. Intrexon better DNA. APHIS-Am I Regulated. https://www.aphis.usda.gov/biotechnology/downloads/reg_loi/18-243-01_a4_air_cbidel.pdf.
98. Alagoz Y, Gurkok T, Zhang B, Unver T. Manipulating the Biosynthesis of Bioactive Compound Alkaloids for Next-Generation Metabolic Engineering in Opium Poppy Using CRISPR-Cas 9 Genome Editing Technology. Sci Rep. 2016;6:30910–8. doi:10.1038/srep30910.
99. United States Department of Agriculture (USDA). 2014. https://www.aphis.usda.gov/biotechnology/downloads/reg_loi/cellectis_air_fad2k0_soy_cbidel.pdf. Accessed 25 Aug 2018.

100. United States Department of Agriculture (USDA). 2015. https://www.aphis.usda.gov/biotechnology/downloads/reg_loi/15-071-01air.pdf. Accessed 25 Aug 2018.
101. Demorest ZL, Coffman A, Baltes NJ, Stoddard TJ, Clasen BM, Luo S, et al. Direct stacking of sequence-specific nuclease-induced mutations to produce high oleic and low linolenic soybean oil. BMC Plant Biol. 2016;16:225–32. doi:10.1186/s12870-016-0906-1.
102. Haun W, Coffman A, Clasen BM, Demorest ZL, Lowy A, Ray E, et al. Improved soybean oil quality by targeted mutagenesis of the fatty acid desaturase 2 gene family. Plant Biotechnol J. 2014;12:934–40. doi:10.1111/pbi.12201.
103. Al Amin N, Ahmad N, Wu N, Pu X, Ma T, Du Y, et al. CRISPR-Cas9 mediated targeted disruption of FAD2-2 microsomal omega-6 desaturase in soybean (Glycine max.L) 2019. doi:10.1186/s12896-019-0501-2.
104. Klap C, Yeshayahou E, Bolger AM, Arazi T, Gupta SK, Shabtai S, et al. Tomato

facultative parthenocarpy results from SlAGAMOUS-LIKE 6 loss of function. Plant Biotechnol J. 2017;15:634–47. doi:10.1111/pbi.12662.
105. Ueta R, Abe C, Watanabe T, Sugano SS, Ishihara R, Ezura H, et al. Rapid breeding of parthenocarpic tomato plants using CRISPR/Cas9. Sci Rep. 2017;7:507. doi:10.1038/s41598-017-00501-4.
106. Lee J, Nonaka S, Takayama M, Ezura H. Utilization of a Genome-Edited Tomato (Solanum lycopersicum) with High Gamma Aminobutyric Acid Content in Hybrid Breeding. J. Agric. Food Chem. 2018;66:963–71. doi:10.1021/acs.jafc.7b05171.
107. Nonaka S, Arai C, Takayama M, Matsukura C, Ezura H. Efficient increase of ɣaminobutyric
acid (GABA) content in tomato fruits by targeted mutagenesis. Sci Rep. 2017;7:7057. doi:10.1038/s41598-017-06400-y.
108. Li X, Wang Y, Chen S, Tian H, Fu D, Zhu B, et al. Lycopene Is Enriched in Tomato Fruit by CRISPR/Cas9-Mediated Multiplex Genome Editing. Front. Plant Sci. 2018;9:179. doi:10.3389/fpls.2018.00559.
109. Yu Q-h, Wang B, Li N, Tang Y, Yang S, Yang T, et al. CRISPR/Cas9-induced Targeted Mutagenesis and Gene Replacement to Generate Long-shelf Life Tomato Lines. Sci Rep. 2017;7:818. doi:10.1038/s41598-017-12262-1.
110. Abe F, Haque E, Hisano H, Tanaka T, Kamiya Y, Mikami M, et al. Genome-Edited Triple-Recessive Mutation Alters Seed Dormancy in Wheat. Cell Rep. 2019;28:1362-1369.e4. doi:10.1016/j.celrep.2019.06.090.
111. United States Department of Agriculture (USDA). 2017. https://www.aphis.usda.gov/biotechnology/downloads/reg_loi/17-038-01_air_inquiry_cbidel.pdf. Accessed 25 Aug 2018.
112. Sánchez-León S, Gil-Humanes J, Ozuna CV, Giménez MJ, Sousa C, Voytas DF, Barro F. Low-gluten, nontransgenic wheat engineered with CRISPR/Cas9 2018. doi:10.1111/pbi.12837.
113. Tripathi JN, Ntui VO, Ron M, Muiruri SK, Britt A, Tripathi L. CRISPR/Cas9 editing of endogenous banana streak virus in the B genome of Musa spp. overcomes a major challenge in banana breeding 2019. doi:10.1038/s42003-019-0288-7.
114. Zhang ZN, Ge XY, Luo XL, Wang P, Fan Q, Hu G, et al. Simultaneous Editing of Two Copies of Gh14-3-3d Confers Enhanced Transgene-Clean Plant Defense Against Verticillium dahliae in Allotetraploid Upland Cotton. Front Plant Sci. 2018;9.
115. Kis A, Hamar E, Tholt G, Ban R, Havelda Z. Creating highly efficient resistance against wheat dwarf virus in barley by employing CRISPR/Cas9 system. 1467-7644. 2019;17:1004–6. doi:10.1111/pbi.13077.
116. Jia H, Orbovic V, Jones JB, Wang N. Modification of the PthA4 effector binding elements in Type I CsLOB1 promoter using Cas9/sgRNA to produce transgenic Duncan
32
grapefruit alleviating XccΔpthA4:dCsLOB1.3 infection. Plant Biotechnol J. 2016;14:1291–301. doi:10.1111/pbi.12495.
117. Jia H, Zhang Y, Orbović V, Xu J, White FF, Jones JB, Wang N. Genome editing of the disease susceptibility gene CsLOB1 in citrus confers resistance to citrus canker. Plant Biotechnol J. 2017;15:817–23. doi:10.1111/pbi.12677.
118. Jia H, Orbovic V, Wang N. CRISPR-LbCas12a-mediated modification of citrus. 1467-7644 2019. doi:10.1111/pbi.13109.
119. Chandrasekaran J, Brumin M, Wolf D, Leibman D, Klap C, Pearlsman M, et al. Development of broad virus resistance in non-transgenic cucumber using CRISPR/Cas9 technology. Mol Plant Pathol. 2016;17:1140–53. doi:10.1111/mpp.12375.
120. Fister AS, Landherr L, Maximova SN, Guiltinan MJ. Transient Expression of CRISPR/Cas9 Machinery Targeting TcNPR3 Enhances Defense Response in Theobroma

cacao. Front. Plant Sci. 2018;9:47. doi:10.3389/fpls.2018.00268.
121. Makhotenko AV, Khromov AV, Snigir EA, Makarova SS, Makarov VV, Suprunova TP, et al. Functional Analysis of Coilin in Virus Resistance and Stress Tolerance of Potato Solanum tuberosum using CRISPR-Cas9 Editing. Dokl Biochem Biophys. 2019;484:88–91. doi:10.1134/S1607672919010241.
122. Zhan X, Zhang F, Zhong Z, Chen R, Wang Y, Chang L, et al. Generation of virusresistant
potato plants by RNA genome targeting 2019. doi:10.1111/pbi.13102.
123. United States Department of Agriculture (USDA). 2017. https://www.aphis.usda.gov/biotechnology/downloads/reg_loi/17-076-01_air_inquiry_cbidel.pdf. Accessed 25 Aug 2018.
124. Gomez MA, Lin ZD, Moll T, Chauhan RD, Hayden L, Renninger K, et al. Simultaneous CRISPR/Cas9-mediated editing of cassava eIF4E isoforms nCBP-1 and nCBP-2 reduces cassava brown streak disease symptom severity and incidence. 1467-7644. 2019;17:421–34. doi:10.1111/pbi.12987.
125. Peng A, Chen S, Lei T, Xu L, He Y, Wu L, et al. Engineering canker-resistant plants through CRISPR/Cas9-targeted editing of the susceptibility gene CsLOB1 promoter in citrus. Plant Biotechnol J. 2017;15:1509–19. doi:10.1111/pbi.12733.
126. Sun QF, Lin L, Liu DX, Wu DW, Fang YJ, Wu J, Wang YP. CRISPR/Cas9-Mediated Multiplex Genome Editing of the BnWRKY11 and BnWRKY70 Genes in Brassica napus L. Int J Mol Sci. 2018;19.
127. Wang F, Wang C, Liu P, Lei C, Hao W, GAO Y, et al. Enhanced Rice Blast Resistance by CRISPR/Cas9-Targeted Mutagenesis of the ERF Transcription Factor Gene OsERF922. PLoS ONE. 2016;11:e0154027. doi:10.1371/journal.pone.0154027.
128. Zhou XC, Jiang GH, Yang LW, Qiu L, He P, Nong CX, et al. Gene diagnosis and targeted breeding for blast-resistant Kongyu 131 without changing regional adaptability. J Genet Genomics. 2018;45:539–47. doi:10.1016/j.jgg.2018.08.003.
129. Li S, Shen L, Hu P, Liu Q, Zhu X, Qian Q, et al. Developing disease-resistant thermosensitive male sterile rice by multiplex gene editing 2019. doi:10.1111/jipb.12774.
130. Zhou J, Peng Z, Long J, Sosso D, Liu B, Eom J-S, et al. Gene targeting by the TAL effector PthXo2 reveals cryptic resistance gene for bacterial blight of rice. Plant J. 2015;82:632–43. doi:10.1111/tpj.12838.
131. Blanvillain-Baufumé S, Reschke M, Solé M, Auguy F, Doucoure H, Szurek B, et al. Targeted promoter editing for rice resistance to Xanthomonas oryzae pv. oryzae reveals differential activities for SWEET14-inducing TAL effectors. Plant Biotechnol J. 2017;15:306–17. doi:10.1111/pbi.12613.
132. Li T, Liu B, Spalding MH, Weeks DP, Yang B. High-efficiency TALEN-based gene editing produces disease-resistant rice. Nat Biotechnol. 2012;30:390–2. doi:10.1038/nbt.2199.

133. United States Department of Agriculture (USDA). 2014. https://www.aphis.usda.gov/biotechnology/downloads/reg_loi/air_isu_ting_rice.pdf. Accessed 25 Aug 2018.
134. Wang J, Tian D, Gu K, Yang X, Wang L, Zeng X, Yin Z. Induction of Xa10-like Genes in Rice Cultivar Nipponbare Confers Disease Resistance to Rice Bacterial Blight. Mol Plant Microbe Interact. 2017;30:466–77. doi:10.1094/MPMI-11-16-0229-R.
135. Xie C, Zhang G, Zhang Y, Song X, Guo H, Chen X, Fang R. SRWD1, a novel target gene of DELLA and WRKY proteins, participates in the development and immune response of rice (Oryza sativa L.). Science Bulletin. 2017;62:1639–48. doi:10.1016/j.scib.2017.12.002.
136. Zhou X, Liao H, Chern M, Yin J, Chen Y, Wang J, et al. Loss of function of a rice

TPR-domain RNA-binding protein confers broad-spectrum disease resistance. Proc Natl Acad Sci U S A. 2018;115:3174–9. doi:10.1073/pnas.1705927115.
137. Cai L, Cao Y, Xu Z, Ma W, Zakria M, Zou L, et al. A Transcription Activator-Like Effector Tal7 of Xanthomonas oryzae pv. oryzicola Activates Rice Gene Os09g29100 to Suppress Rice Immunity. Sci Rep. 2017;7:5089. doi:10.1038/s41598-017-04800-8.
138. Macovei A, Sevilla NR, Cantos C, Jonson GB, Slamet-Loedin I, Čermák T, et al. Novel alleles of rice eIF4G generated by CRISPR/Cas9-targeted mutagenesis confer resistance to Rice tungro spherical virus. Plant Biotechnol J. 2018;47:417. doi:10.1111/pbi.12927.
139. Zhang T, Zhao YL, Ye JJ, Cao X, Xu CH, Chen B, et al. Establishing CRISPR/Cas13a immune system conferring RNA virus resistance in both dicot and monocot plants. 1467-7644. 2019;17:1185–7. doi:10.1111/pbi.13095.
140. Nekrasov V, Wang C, Win J, Lanz C, Weigel D, Kamoun S. Rapid generation of a transgene-free powdery mildew resistant tomato by genome deletion. Sci Rep. 2017;7:482. doi:10.1038/s41598-017-00578-x.
141. Mahfouz M, Tashkandi M, Ali Z, Aljedaani F, Shami A. Engineering resistance against Tomato yellow leaf curl virus via the CRISPR/Cas9 system in tomato 2017. doi:10.1101/237735.
142. Toledo Thomazella DP de, Brail Q, Dahlbeck D, Staskawicz BJ. CRISPR-Cas9 mediated mutagenesis of a DMR6 ortholog in tomato confers broad-spectrum disease resistance. 2016:1–23. doi:10.1101/064824.
143. Ortigosa A, Gimenez-Ibanez S, Leonhardt N, Solano R. Design of a bacterial speck resistant tomato by CRISPR/Cas9-mediated editing of SlJAZ2. 1467-7644. 2019;17:665–73. doi:10.1111/pbi.13006.
144. Wang X, Tu M, Wang D, Liu J, Li Y, Li Z, et al. CRISPR/Cas9-mediated efficient targeted mutagenesis in grape in the first generation. Plant Biotechnol J. 2018;16:844–55. doi:10.1111/pbi.12832.
145. Wang Y, Cheng X, Shan Q, Zhang Y, Liu J, Gao C, Qiu J-L. Simultaneous editing of three homoeoalleles in hexaploid bread wheat confers heritable resistance to powdery mildew. Nat Biotechnol. 2014;32:947–51. doi:10.1038/nbt.2969.
146. Zhang Y, Bai Y, Wu G, Zou S, Chen Y, Gao C, Tang D. Simultaneous modification of three homoeologs of TaEDR1 by genome editing enhances powdery mildew resistance in wheat. Plant J. 2017;91:714–24. doi:10.1111/tpj.13599.
147. D'Halluin K, Vanderstraeten C, van Hulle J, Rosolowska J, van den Brande I, Pennewaert A, et al. Targeted molecular trait stacking in cotton through targeted doublestrand break induction. Plant Biotechnol J. 2013;11:933–41. doi:10.1111/pbi.12085.
148. Sauer NJ, Narváez-Vásquez J, Mozoruk J, Miller RB, Warburg ZJ, Woodward MJ, et al. Oligonucleotide-Mediated Genome Editing Provides Precision and Function to Engineered Nucleases and Antibiotics in Plants. Plant Physiol. 2016;170:1917–28. doi:10.1104/pp.15.01696.

149. Butler NM, Baltes NJ, Voytas DF, Douches DS. Geminivirus-Mediated Genome Editing in Potato (Solanum tuberosum L.) Using Sequence-Specific Nucleases. Front Plant Sci. 2016;7:1–13. doi:10.3389/fpls.2016.01045.
150. Svitashev S, Young JK, Schwartz C, Gao H, Falco SC, Cigan AM. Targeted Mutagenesis, Precise Gene Editing, and Site-Specific Gene Insertion in Maize Using Cas9 and Guide RNA. Plant Physiol. 2015;169:931–45. doi:10.1104/pp.15.00793.
151. Svitashev S, Schwartz C, Lenderts B, Young JK, Mark Cigan A. Genome editing in maize directed by CRISPR-Cas9 ribonucleoprotein complexes. Nat Commun. 2016;7:1–7. doi:10.1038/ncomms13274.
152. Ainley WM, Sastry-Dent L, Welter ME, Murray MG, Zeitler B, Amora R, et al. Trait

stacking via targeted genome editing. Plant Biotechnol J. 2013;11:1126–34. doi:10.1111/pbi.12107.
153. Zhu T, Peterson DJ, Tagliani L, St. Clair G, Baszczynski CL, Bowen B. Targeted manipulation of maize genes in vivo using chimeric RNA/DNA oligonucleotides. Proc. Natl. Acad. Sci. 1999;96:8768–73. doi:10.1073/pnas.96.15.8768.
154. Zhu T, Mettenburg K, Peterson DJ, Tagliani L, Baszczynski CL. Engineering herbicide-resistant maize using chimeric RNA/DNA oligonucleotides. Nat Biotechnol. 2000;18:555–8. doi:10.1038/75435.
155. Hummel AW, Chauhan RD, Cermak T, Mutka AM, Vijayaraghavan A, Boyher A, et al. Allele exchange at the EPSPS locus confers glyphosate tolerance in cassava. 1467-7644. 2018;16:1275–82. doi:10.1111/pbi.12868.
156. Gocal GFW, Schöpke C, Beetham PR. Oligo-Mediated Targeted Gene Editing. In: Advances in New Technology for Targeted Modification of Plant Genomes; 2015. p. 73–89. doi:10.1007/978-1-4939-2556-8_5.
157. Ruiter R, van den Brande I, Stals E, Delauré S, Cornelissen M, D'Halluin K. Spontaneous mutation frequency in plants obscures the effect of chimeraplasty. Plant Mol Biol. 2003;53:675–89. doi:10.1023/b:plan.0000019111.96107.01.
158. Li J, Meng X, Zong Y, Chen K, Zhang H, Liu J, et al. Gene replacements and insertions in rice by intron targeting using CRISPR-Cas9. NPLANTS. 2016;2:1–6. doi:10.1038/nplants.2016.139.
159. Sun Y, Zhang X, Wu C, He Y, Ma Y, Hou H, et al. Engineering Herbicide-Resistant Rice Plants through CRISPR/Cas9-Mediated Homologous Recombination of Acetolactate Synthase. Mol Plant. 2016;9:628–31. doi:10.1016/j.molp.2016.01.001.
160. Wang M, Liu Y, Zhang C, Liu J, Liu X, Wang L, et al. Gene editing by cotransformation of TALEN and chimeric RNA/DNA oligonucleotides on the rice OsEPSPS gene and the inheritance of mutations. PLoS ONE. 2015;10:e0122755. doi:10.1371/journal.pone.0122755.
161. Okuzaki A, Toriyama K. Chimeric RNA/DNA oligonucleotide-directed gene targeting in rice. Plant Cell Rep. 2004;22:509–12. doi:10.1007/s00299-003-0698-2.
162. Shimatani Z, Kashojiya S, Takayama M, Terada R, Arazoe T, Ishii H, et al. Targeted base editing in rice and tomato using a CRISPR-Cas9 cytidine deaminase fusion. Nat Biotechnol. 2017;35:441–3. doi:10.1038/nbt.3833.
163. Shimatani Z, Fujikura U, Ishii H, Matsui Y, Suzuki M, Ueke Y, et al. Inheritance of co-edited genes by CRISPR-based targeted nucleotide substitutions in rice. Plant Physiol Biochem 2018. doi:10.1016/j.plaphy.2018.04.028.
164. Li C, Zong Y, Wang YP, Jin S, Zhang DB, Song QN, et al. Expanded base editing in rice and wheat using a Cas9-adenosine deaminase fusion. Genome Biol. 2018;19.
165. Butt H, Eid A, Ali Z, Atia MAM, Mokhtar MM, Hassan N, et al. Efficient CRISPR/Cas9-Mediated Genome Editing Using a Chimeric Single-Guide RNA Molecule. Front Plant Sci. 2017;8:1441. doi:10.3389/fpls.2017.01441.

166. Li Z, Liu Z-B, Xing A, Moon BP, Koellhoffer JP, Huang L, et al. Cas9-Guide RNA Directed Genome Editing in Soybean. Plant Physiol. 2015;169:960–70. doi:10.1104/pp.15.00783.
167. Chilcoat D, Liu Z-B, Sander J. Use of CRISPR/Cas9 for Crop Improvement in Maize and Soybean. Prog Mol Biol Transl Sci. 2017;149:27–46. doi:10.1016/bs.pmbts.2017.04.005.
168. Danilo B, Perrot L, Mara K, Botton E, Nogue F, Mazier M. Efficient and transgenefree gene targeting using Agrobacterium-mediated delivery of the CRISPR/Cas9 system in tomato. Plant Cell Rep. 2019;38:459–62. doi:10.1007/s00299-019-02373-6.

169. Tian SW, Jiang LJ, Cui XX, Zhang J, Guo SG, Li MY, et al. Engineering herbicideresistant
watermelon variety through CRISPR/Cas9-mediated base-editing. Plant Cell Rep. 2018;37:1353–6. doi:10.1007/s00299-018-2299-0.
170. Zhang R, Liu JX, Chai ZZ, Chen S, Bai Y, Zong Y, et al. Generation of herbicide tolerance traits and a new selectable marker in wheat using base editing. Nat Plants. 2019;5:480–5. doi:10.1038/s41477-019-0405-0.
171. Andersson M, Turesson H, Nicolia A, Fält A-S, Samuelsson M, Hofvander P. Efficient targeted multiallelic mutagenesis in tetraploid potato (Solanum tuberosum) by transient CRISPR-Cas9 expression in protoplasts. Plant Cell Rep. 2017;36:117–28. doi:10.1007/s00299-016-2062-3.
172. Veillet F, Chauvin L, Kermarrec MP, Sevestre F, Merrer M, Terret Z, et al. The Solanum tuberosum GBSSI gene: a target for assessing gene and base editing in tetraploid potato 2019. doi:10.1007/s00299-019-02426-w.
173. Kusano H, Ohnuma M, Mutsuro-Aoki H, Asahi T, Ichinosawa D, Onodera H, et al. Establishment of a modified CRISPR/Cas9 system with increased mutagenesis frequency using the translational enhancer dMac3 and multiple guide RNAs in potato. Sci Rep-Uk. 2018;8.
174. Wieghaus A, Prufer D, Gronover CS. Loss of function mutation of the Rapid Alkalinization Factor (RALF1)-like peptide in the dandelion Taraxacum koksaghyz entails a high-biomass taproot phenotype. Plos One. 2019;14.
175. Park J-J, Yoo CG, Flanagan A, Pu Y, Debnath S, Ge Y, et al. Defined tetra-allelic gene disruption of the 4-coumarate:coenzyme A ligase 1 (Pv4CL1) gene by CRISPR/Cas9 in switchgrass results in lignin reduction and improved sugar release. Biotechnol Biofuels. 2017;10:284. doi:10.1186/s13068-017-0972-0.
176. Schachtsiek J, Stehle F. Nicotine-free, Non-transgenic Tobacco (Nicotianatabacuml.) Edited by CRISPR-Cas9. 1467-7644 2019. doi:10.1111/pbi.13193.
177. Jung JH, Altpeter F. TALEN mediated targeted mutagenesis of the caffeic acid Omethyltransferase
in highly polyploid sugarcane improves cell wall composition for production of bioethanol. Plant Mol Biol. 2016;92:131–42. doi:10.1007/s11103-016-0499-y.
178. Kannan B, Jung JH, Moxley GW, Lee S-M, Altpeter F. TALEN-mediated targeted mutagenesis of more than 100 COMT copies/alleles in highly polyploid sugarcane improves saccharification efficiency without compromising biomass yield. Plant Biotechnol J. 2018;16:856–66. doi:10.1111/pbi.12833.
179. Njuguna E, Coussens G, Aesaert S, Neyt P, Anami S, van Lijsebettens M. Modulation of energy homeostasis in maize and Arabidopsis to develop lines tolerant to drought, genotoxic and oxidative stresses. AF 2018. doi:10.21825/af.v30i2.8080.
180. Shi J, Gao H, Wang H, Lafitte HR, Archibald RL, Yang M, et al. ARGOS8 variants generated by CRISPR-Cas9 improve maize grain yield under field drought stress conditions. Plant Biotechnol J. 2017;15:207–16. doi:10.1111/pbi.12603.

181. Duan Y-B, Li J, Qin R-Y, Xu R-F, Li H, Yang Y-C, et al. Identification of a regulatory element responsible for salt induction of rice OsRAV2 through ex situ and in situ promoter analysis. Plant Mol Biol. 2016;90:49–62. doi:10.1007/s11103-015-0393-z.
182. Zhang AN, Liu Y, Wang FM, Li TF, Chen ZH, Kong DY, et al. Enhanced rice salinity tolerance via CRISPR/Cas9-targeted mutagenesis of the OsRR22 gene. Mol Breeding. 2019;39.
183. United States Department of Agriculture (USDA). 2017.
https://www.aphis.usda.gov/biotechnology/downloads/reg_loi/17-219-01_air_inquiry.pdf.

Accessed 25 Aug 2018.
184. Kim D, Alptekin B, Budak H. CRISPR/Cas9 genome editing in wheat. Funct Integr Genomics. 2018;18:31–41. doi:10.1007/s10142-017-0572-x.
185. Ma CF, Zhu CZ, Zheng M, Liu MC, Zhang DJ, Liu BL, et al. CRISPR/Cas9-mediated multiple gene editing in Brassica oleracea var. capitata using the endogenous tRNAprocessing system. Hortic Res-England. 2019;6.
186. United States Department of Agriculture (USDA). 2010. https://www.aphis.usda.gov/biotechnology/downloads/reg_loi/DOW_Email_%20to_Susan_%20Kohler_032010.pdf. Accessed 25 Aug 2018.
187. Shukla VK, Doyon Y, Miller JC, DeKelver RC, Moehle EA, Worden SE, et al. Precise genome modification in the crop species Zea mays using zinc-finger nucleases. Nature. 2009;459:437–41. doi:10.1038/nature07992.
188. Lemmon ZH, Reem NT, Dalrymple J, Soyk S, Swartwood KE, Rodriguez-Leal D, et al. Rapid improvement of domestication traits in an orphan crop by genome editing. Nat Plants. 2018;4:766–70. doi:10.1038/s41477-018-0259-x.
189. Wang C, Liu Q, Shen Y, Hua YF, Wang JJ, Lin JR, et al. Clonal seeds from hybrid rice by simultaneous genome engineering of meiosis and fertilization genes. Nat Biotechnol. 2019;37:283-+. doi:10.1038/s41587-018-0003-0.
190. Li TD, Yang XP, Yu Y, Si XM, Zhai XW, Zhang HW, et al. Domestication of wild tomato is accelerated by genome editing. Nat Biotechnol. 2018;36:1160-+. doi:10.1038/nbt.4273.
191. Zsögön A, ?ermák T, Naves ER, Notini MM, Edel KH, Weinl S, et al. De novo domestication of wild tomato using genome editing 2018. doi:10.1038/nbt.4272.
192. Ye MW, Peng Z, Tang D, Yang ZM, Li DW, Xu YM, et al. Generation of selfcompatible diploid potato by knockout of S-RNase. Nat Plants. 2018;4:651–4. doi:10.1038/s41477-018-0218-6.
193. Enciso-Rodriguez F, Manrique-Carpintero NC, Nadakuduti SS, Buell CR, Zarka D, Douches D. Overcoming Self-Incompatibility in Diploid Potato Using CRISPR-Cas9. Front Plant Sci. 2019;10.
194. Xie K, Wu S, Li Z, Zhou Y, Zhang D, Dong Z, et al. Map-based cloning and characterization of Zea mays male sterility33 (ZmMs33) gene, encoding a glycerol-3-phosphate acyltransferase. Theor Appl Genet. 2018;131:1363–78. doi:10.1007/s00122-018-3083-9.
195. Li J, Zhang H, Si X, Tian Y, Chen K, Liu J, et al. Generation of thermosensitive malesterile
maize by targeted knockout of the ZmTMS5 gene. J Genet Genomics. 2017;44:465–8. doi:10.1016/j.jgg.2017.02.002.
196. Chen RR, Xu QL, Liu Y, Zhang JJ, Ren DT, Wang GY, Liu YJ. Generation of Transgene-Free Maize Male Sterile Lines Using the CRISPR/Cas9 System. Front Plant Sci. 2018;9.
197. Kelliher T, Starr D, Richbourg L, Chintamanani S, Delzer B, Nuccio ML, et al. MATRILINEAL, a sperm-specific phospholipase, triggers maize haploid induction. Nature. 2017;542:105–9. doi:10.1038/nature20827.

198. Kelliher T, Starr D, Su XJ, Tang GZ, Chen ZY, Carter J, et al. One-step genome editing of elite crop germplasm during haploid induction. Nat Biotechnol. 2019;37:287-+. doi:10.1038/s41587-019-0038-x.
199. Wang BB, Zhu L, Zhao BB, Zhao YP, Xie YR, Zheng ZG, et al. Development of a Haploid-Inducer Mediated Genome Editing System for Accelerating Maize Breeding. Mol Plant. 2019;12:597–602. doi:10.1016/j.molp.2019.03.006.
200. Zhong Y, Liu C, Qi X, Jiao Y, Wang D, Wang Y, et al. Mutation of ZmDMP enhances

haploid induction in maize 2019. doi:10.1038/s41477-019-0443-7.
201. Lee S-K, Eom J-S, Hwang S-K, Shin D, An G, Okita TW, Jeon J-S. Plastidic phosphoglucomutase and ADP-glucose pyrophosphorylase mutants impair starch synthesis in rice pollen grains and cause male sterility. J Exp Bot. 2016;67:5557–69. doi:10.1093/jxb/erw324.
202. Li Q, Zhang D, Chen M, Liang W, Wei J, Qi Y, Yuan Z. Development of japonica Photo-Sensitive Genic Male Sterile Rice Lines by Editing Carbon Starved Anther Using CRISPR/Cas9. J Genet Genomics. 2016;43:415–9. doi:10.1016/j.jgg.2016.04.011.
203. Shi QS, Wang KQ, Li YL, Zhou L, Xiong SX, Han Y, et al. OsPKS1 is required for sexine layer formation, which shows functional conservation between rice and Arabidopsis. 0168-9452. 2018;277:145–54. doi:10.1016/j.plantsci.2018.08.009.
204. Xie Y, Niu B, Long Y, Li G, Tang J, Zhang Y, et al. Suppression or knockout of SaF/SaM overcomes the Sa-mediated hybrid male sterility in rice. J Integr Plant Biol. 2017;59:669–79. doi:10.1111/jipb.12564.
205. Zhou H, He M, Li J, Chen L, Huang Z, Zheng S, et al. Development of Commercial Thermo-sensitive Genic Male Sterile Rice Accelerates Hybrid Rice Breeding Using the CRISPR/Cas9-mediated TMS5 Editing System. Sci Rep. 2016;6:1–12. doi:10.1038/srep37395.
206. Zou T, He Z, Qu L, Liu M, Zeng J, Liang Y, et al. Knockout of OsACOS12 caused male sterility in rice. Mol Breeding. 2017;37:437. doi:10.1007/s11032-017-0722-9.
207. Zou T, Xiao Q, Li W, Luo T, Yuan G, He Z, et al. OsLAP6/OsPKS1, an orthologue of Arabidopsis PKSA/LAP6, is critical for proper pollen exine formation. Rice (N Y). 2017;10:615. doi:10.1186/s12284-017-0191-0.
208. Barman HN, Sheng ZH, Fiaz S, Zhong M, Wu YW, Cai YC, et al. Generation of a new thermo-sensitive genic male sterile rice line by targeted mutagenesis of TMS5 gene through CRISPR/Cas9 system. Bmc Plant Biol. 2019;19.
209. Shen L, Dong GJ, Zhang Y, Hu GC, Zhang Q, Hu GL, et al. Rapid Creation of New Photoperiod-/Thermo-Sensitive Genic Male-Sterile Rice Materials by CRISPR/Cas9 System. Rice Sci. 2019;26:129–32. doi:10.1016/j.rsci.2018.12.006.
210. Khanday I, Skinner D, Yang B, Mercier R, Sundaresan V. A male-expressed rice embryogenic trigger redirected for asexual propagation through seeds. Nature. 2019;565:91-+. doi:10.1038/s41586-018-0785-8.
211. Singh M, Kumar M, Albertsen MC, Young JK, Cigan AM. Concurrent modifications in the three homeologs of Ms45 gene with CRISPR-Cas9 lead to rapid generation of male sterile bread wheat (Triticum aestivum L.). Plant Mol Biol. 2018;97:371–83. doi:10.1007/s11103-018-0749-2.
212. Okada A, Arndell T, Borisjuk N, Sharma N, Watson-Haigh NS, Tucker EJ, et al. CRISPR/Cas9-mediated knockout of Ms1 enables the rapid generation of male-sterile hexaploid wheat lines for use in hybrid seed production 2019. doi:10.1111/pbi.13106.
213. Watanabe K, Kobayashi A, Endo M, Sage-Ono K, Toki S, Ono M. CRISPR/Cas9-mediated mutagenesis of the dihydroflavonol-4-reductase-B (DFR-B) locus in the Japanese morning glory Ipomoea (Pharbitis) nil. Sci Rep. 2017;7:10028. doi:10.1038/s41598-017-10715-1
214. Watanabe K, Oda-Yamamizo C, Sage-Ono K, Ohmiya A, Ono M. Alteration of flower colour in Ipomoea nil through CRISPR/Cas9-mediated mutagenesis of carotenoid cleavage dioxygenase 4. Transgenic Res. 2017;27:25–38. doi:10.1007/s11248-017-0051-0.
215. Shibuya K, Watanabe K, Ono M. CRISPR/Cas9-mediated mutagenesis of the EPHEMERAL1 locus that regulates petal senescence in Japanese morning glory. Plant Physiol Biochem 2018. doi:10.1016/j.plaphy.2018.04.036.
216. Kui L, Chen H, Zhang W, He S, Xiong Z, Zhang Y, et al. Building a Genetic

Manipulation Tool Box for Orchid Biology: Identification of Constitutive Promoters and Application of CRISPR/Cas9 in the Orchid, Dendrobium officinale. Front Plant Sci. 2016;7:2036. doi:10.3389/fpls.2016.02036.
217. Xu J, Kang BC, Naing AH, Bae SJ, Kim JS, Kim H, Kim CK. CRISPR/Cas9-mediated editing of 1-aminocyclopropane-1-carboxylate oxidase1 (ACO1) enhances Petunia flower longevity. 1467-7644 2019. doi:10.1111/pbi.13197.
218. Xie X, Qin G, Si P, Luo Z, Gao J, Chen X, et al. Analysis of Nicotiana tabacum PIN genes identifies NtPIN4 as a key regulator of axillary bud growth. Physiol Plant. 2017;160:222–39. doi:10.1111/ppl.12547.
219. Nishihara M, Higuchi A, Watanabe A, Tasaki K. Application of the CRISPR/Cas9 system for modification of flower color in Torenia fournieri. BMC Plant Biol. 2018;18:331. doi:10.1186/s12870-018-1539-3.
